# Covalent Organic Frameworks and 2D Materials Hybrids: Synthesis Strategies, Properties Enhancements, and Future Directions

**DOI:** 10.1002/smll.202410544

**Published:** 2024-12-29

**Authors:** Cataldo Valentini, Verónica Montes‐García, Dawid Pakulski, Paolo Samorì, Artur Ciesielski

**Affiliations:** ^1^ Center for Advanced Technologies Adam Mickiewicz University Uniwersytetu Poznańskiego 10 Poznań 61‐614 Poland; ^2^ Faculty of Chemistry Adam Mickiewicz University Uniwersytetu Poznańskiego 8 Poznań 61‐614 Poland; ^3^ Université de Strasbourg CNRS ISIS UMR 7006 8 allée Gaspard Monge Strasbourg 67000 France

**Keywords:** 2D materials, advanced materials, covalent organic frameworks, hybrid materials, porous materials

## Abstract

Covalent organic frameworks (COFs) are highly porous, thermally and chemically stable organic polymers. Their high porosity, crystallinity, and adjustable properties make them suitable for numerous applications. However, COFs encounter critical challenges, such as their difficult processability, self‐stacking propensity, low electrical conductivity, pore blockage which limits their ionic conductivity, and high recombination rates of photoinduced electrons and holes. To overcome these issues, the hybridization of COFs with 2D materials (2DMs) has proven to be an effective strategy. 2DMs including graphene‐like materials, transition metal dichalcogenides, and MXenes are particularly advantageous because of their unique physicochemical properties, such as exceptional electrical and optical characteristics, and mechanical resilience. Over the past decade, significant research efforts have been focused on hybrid 2DMs‐COFs materials. These hybrids leverage the strengths of both materials, making them suitable for advanced applications. This Review highlights the latest advancements in 2DM‐COF hybrids, examining the physicochemical strengths and weaknesses of the pristine materials, together with the synergistic benefits of their hybridization. Moreover, it emphasizes their most remarkable applications in chemical sensing, catalysis, energy storage, adsorption and filtration, and as anticorrosion agents. Finally, it discusses future challenges and opportunities in the development of 2DM‐COFs for new disruptive technologies.

## Graphical abstract

This review presents the current state of 2D materials (2DMs) ‐ covalent organic frameworks (COFs) hybrids, focusing on their physicochemical properties, synthesis methods, and applications in sensing, catalysis, energy storage, and filtration. It outlines the strengths and weaknesses of 2DMs and COFs, highlights synergistic benefits from hybridization, and discusses future challenges and opportunities for technological advancements.

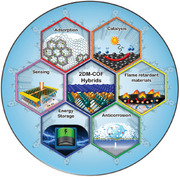

## 1 Introduction

First synthesized in 2005 by Yaghi et al. covalent organic frameworks (COFs) have emerged as a prominent class of thermally and chemically stable organic porous polymers characterized by high porosity and crystallinity.^[^ ^1^, ^2^, ^3^, ^4^, ^5^, ^6^ ^]^ The design of COFs’ structures provides extensive chemical engineering opportunities, enabling the formation of both 1D, 2D, and 3D shape persistent architectures, and also allowing precise control over surface areas, pore sizes, and crystal structures.^[^ ^7^, ^8^, ^9^, ^10^ ^]^ 1D COFs generally feature linear or chain‐like architectures, characterized by unique optical properties arising from their extended π‐conjugation and non‐covalent interactions.^[^ ^11^ ^]^ Similarly, 2D COFs form layered structures that predominantly stack in a face‐to‐face manner, driven by strong π–π interactions between the monomeric units. This supramolecular arrangement enhances interlayer charge mobility and facilitates the formation of 1D channels, which are beneficial for efficient mass transport.^[^ ^12^ ^]^ In contrast, 3D COFs exhibit intricate 3D architectures that lack π–π interaction and, thereby limiting the short‐range charge transfer between the monomers. Nevertheless, their 3D connectivity often imparts superior porosity and enhanced chemical stability.^[^ ^13^ ^]^

By employing the topological monomer design principles established for similar materials such as metal‐organic frameworks (MOFs), the polymeric topology of COFs can be programmed.^[^ ^1^ ^]^ Highly ordered COFs can be synthesized by harnessing dynamic covalent chemistry (DCC) strategies employing reversible and dynamic covalent bonds, such as imine (C═N),^[^ ^14^ ^]^ imide (O═C─N─C═O),^[^ ^15^, ^16^ ^]^ boroxine (B_3_O_3_)^[^ ^1^, ^17^ ^]^ and boronic ester bonds (O─B─O),^[^ ^18^ ^]^ to allow the generation of COFs under thermodynamic control.^[^ ^19^ ^]^ Due to their remarkable physicochemical properties and robustness, COFs are highly versatile materials suitable for various applications, including chemical sensing, energy storage systems (ESS), catalysis, adsorption and smart coatings,^[^ ^3^, ^8^, ^9^, ^10^, ^20^, ^21^, ^22^, ^23^, ^24^, ^25^, ^26^, ^27^, ^28^, ^29^, ^30^, ^31^, ^32^, ^33^ ^]^ to name a few. During COF synthesis, specific functional groups or active sites can be incorporated into building monomers to enhance their capabilities in sensing, catalysis, electrochemistry, adsorption, and separation. The large surface area of COFs enhances sensitivity in chemical sensing and provides ample space for charge accumulation in energy storage, thereby improving electrochemical performance. Additionally, the unique channel structures and adjustable porosities of COFs facilitate carrier migration, efficient access to active sites, and rapid mass transport.^[^ ^34^, ^35^ ^]^ Furthermore, COFs possess an adjustable bandgap, enabling modulation of their electronic structures, together with an excellent light‐capturing capability in the visible region. These properties, combined with the ease of separation and reuse as heterogeneous photocatalysts, significantly enhance catalytic performance. In the field of energy storage, COFs’ robust structure allows them to withstand repeated charge and discharge cycles, improving long‐term cycling stability. For anticorrosion applications, COFs serve as nanocontainers of inhibitors that can be released upon external stimuli, enabling the fabrication of smart coatings with self‐healing properties. Their high loading capacity and smart release of inhibitors make them effective in suppressing surface corrosion, and their outstanding thermomechanical properties ensure stability in harsh environments and organic polymeric matrices.

Despite their numerous advantages, the use of COFs as advanced materials faces several challenges.^[^ ^7^ ^]^ COFs are typically synthesized as insoluble solid powders through solvothermal methods, leading to anisotropic growth that complicates reliable device integration. In catalysis, drawbacks such as high recombination rates of photogenerated electron and hole pairs, inefficient interlayer conductivity, microporous nature in most cases, and lack of monolithic form hinder mass transport and electrocatalytic activity. For chemical sensing and energy storage applications, low electrical conductivity and low carrier mobility hamper the performance of devices with electrical output and affect charge transfer kinetics, respectively. In adsorption and separation as well as in energy storage applications, the reversibility of dynamic covalent bonds (e.g., imine), in certain experimental conditions, limits practical applications.

Moreover, the staggered supramolecular organization of COF layers can obstruct the pores, impeding mass transfer, reducing the accessibility to (electrochemically)‐active sites, and diminishing surface area and adsorption efficiency. Additionally, for anticorrosion applications, the porous structure of COFs can act as pathways for corrosive agents, potentially weakening their shielding effect against corrosion.

**Figure 1:**
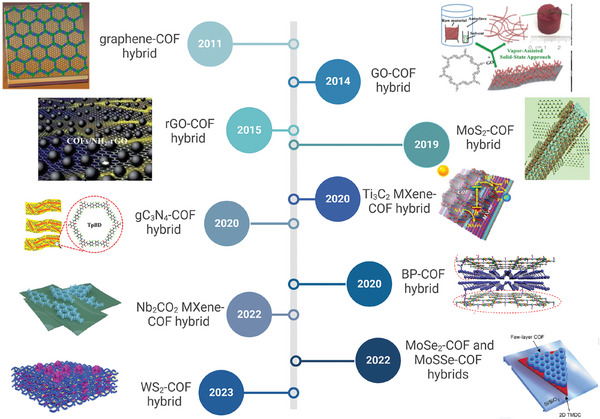
The timeline of development for 2DM‐COF hybrids, highlighting the key milestones marked by the introduction of the earliest examples of these hybrids. Graphical thumbnails Reproduced with permission,^[^ ^36^, ^37^, ^38^, ^39^, ^40^, ^41^, ^42^, ^43^, ^44^ ^]^ respectively Copyright 2011, AAAS (Science); Copyright 2014, Royal Society of Chemistry; Copyright 2015, Royal Society of Chemistry; Copyright 2019, Elsevier Science & Technology Journals; Copyright 2020, American Chemical Society; Copyright 2020, Elsevier Science & Technology Journals; Copyright 2022, American Chemical Society; Copyright 2023, Royal Society of Chemistry; Copyright 2021.

To address these drawbacks, a promising strategy consists of the hybridization of COFs with functional low‐dimensional nanostructures, yielding systems exhibiting properties that are beyond the mere sum of those of the individual components.^[^ ^6^, ^45^, ^46^, ^47^, ^48^ ^]^ Among various low‐dimensional nanostructures, 2D materials (2DMs) are particularly well‐suited due to their unique chemical and physical characteristics, such as high surface‐to‐volume ratios, high sensitivity to environmental changes, and, in some cases, exceptional electrical and optical properties, along with remarkable mechanical resilience and flexibility.^[^ ^2^, ^10^, ^49^, ^50^, ^51^, ^52^, ^53^, ^54^ ^]^ During the last decade (**Figure** **1**), considerable research effort has been dedicated to the synthesis of hybrid materials that combine 2DMs, such as graphene‐related materials (GRM) including graphene (since 2011),^[^ ^36^ ^]^ graphene oxide (GO) (since 2014)^[^ ^37^ ^]^ and reduced graphene oxide (rGO) (since 2015),^[^ ^38^ ^]^ transition metal dichalcogenides (MoS_2_) (since 2019),^[^ ^39^ ^]^ early transition metal carbides/nitrides referred to as MXenes (since 2020),^[^ ^40^ ^]^ graphitic carbon nitride (g‐C_3_N_4_) (since 2020),^[^ ^41^ ^]^ or black phosphorous (BP) (since 2020),^[^ ^42^ ^]^ MoSe_2_ (since 2022),^[^ ^43^ ^]^ WS_2_ (since 2023)^[^ ^44^ ^]^ with COFs, yielding 2DM‐COF hybrids. These combinations result in porous materials with enhanced properties ideal for catalysis,^[^ ^45^, ^55^ ^]^ energy storage,^[^ ^46^, ^56^ ^]^ adsorption,^[^ ^6^, ^48^ ^]^ sensors,^[^ ^6^, ^48^ ^]^ and anticorrosion applications.^[^ ^57^ ^]^ These endeavors are aimed at overcoming the limitations of COFs and 2DMs by enhancing the physicochemical properties of individual components (e.g., surface area and pore structure, stability, conductivity, and wettability), thereby improving their overall performance. Therefore, understanding and controlling the parameters of hybridization is crucial to unlock the full potential of 2DM‐COF hybrids across various technological domains.

With the growing interest in 2DM‐COF hybrids, this Review aims to present the current state of the art in terms of their physicochemical properties, synthetic methodologies, and applications across various fields. The initial section focuses on delineating the strengths and weaknesses of various 2DMs in terms of their physicochemical properties, highlighting the synergistic benefits achieved through their hybridization with COFs. Subsequently, we explore the most promising synthetic approaches for developing such hybrids. The most enlightening examples of 2DM‐COF hybrids are discussed in the third section, emphasizing their applications in chemical sensing, catalysis, energy storage, and adsorption and filtration. Finally, we address future directions by examining the challenges and opportunities inherent in advancing 2DM‐COF hybrids to drive impactful technological innovations across diverse domains.

## 2 Overcoming the Limitations of 2DMs Through Hybridization with COFs

**Figure 2:**
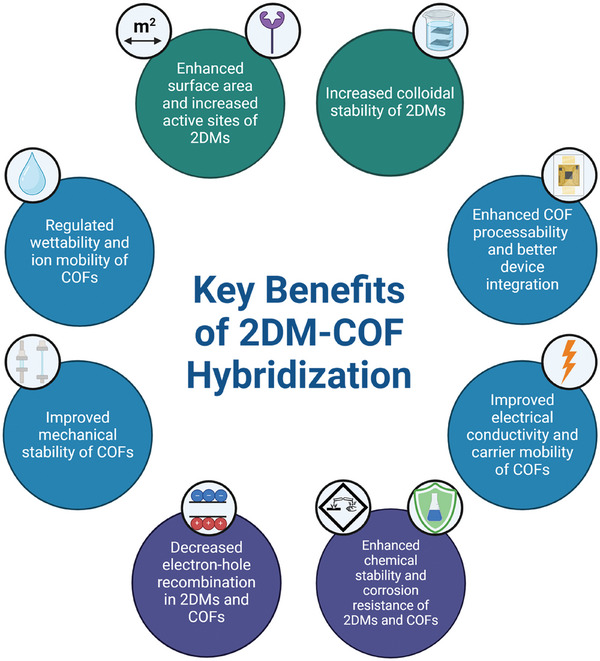
Synergies of 2DM‐COFs hybrids.

Hybridizing 2DMs with COFs has emerged as a powerful strategy to address the intrinsic limitations of both material classes while fostering the emergence of new properties (**Table** **1**, **Figure** **2**). This synergistic approach leverages the unique properties of COFs including high porosity, controllable bandgaps, and tunable chemical characteristics, and combines them with the exceptional electrical, mechanical, and optical properties of 2DMs. These hybrid systems exhibit enhanced physicochemical properties, enabling advanced applications in energy storage, catalysis, adsorption and filtration, sensing, and anticorrosion.

**Table 1:** Summary of the advantages and disadvantages of pristine COFs and 2DMs in various applications.

Material	Advantages	Disadvantages
COFs	Large surface area Δ □ ° ◊ ▪ Adjustable porosity Δ □ ° ◊ ▪ Versatile functionalization Δ □ ° ◊ ▪ Adjustable bandgap Δ □ ° Excellent light‐capturing capability in the visible region □ Ease of separation and reuse □ High chemical stability (depending on the employed linkage) Δ □ ° ◊ ▪ COFs can act as nanocontainers with high loading capacity where molecules can be released upon external stimuli ▪ Outstanding thermomechanical properties (depending on the employed linkage) ▪	Difficult processability Δ ° ▪ Microporous nature is most of the cases Δ □ ° ◊ ▪ Poor blockage due to staggered supramolecular organization of COF layers Δ □ ° ◊ ▪ High recombination rates of photogenerated electron and hole pairs□ Inefficient interlayer conductivity Δ □ ° Low electrical conductivity Δ □ ° Low carrier mobility Δ □ ° Poor chemical stability in certain experimental conditions (depending on the employed linkage) Δ □ ° ◊ ▪
All 2DMs	Large surface area Δ □ ° ◊ ▪ High conductivity (GRMs, 1T phase MoS_2_ or MXenes) Δ □	Self‐agglomeration Δ □ ° ◊ ▪
GRMs	High chemical stability Δ □ ° ◊ ▪ Versatile functionalization Δ □ ° ◊ ▪ Remarkable mechanical properties ▪ Fast response times to changes in their environment Δ □ ◊ ▪	Broad light absorption Δ □ Limited absorption capacity □ High recombination rates of photogenerated electron and hole pairs□
MoS_2_	2H form‐notable electron transfer Δ Tunable band‐gap Δ □ ° High chemical stability Δ □ ° ◊ ▪ Excellent light‐capturing capability □ High carrier mobility Δ □ ° Good mechanical properties ▪ Acts as a barrier for corrosion ▪	Limited sensitivity in chemical sensing Δ Susceptibility to interferences Δ Complex functionalization Δ □ ° ◊ ▪ High recombination rates of photogenerated electron and hole pairs□
MXenes	Abundant active sites Δ □ ° ◊ ▪ Controllable layer spacing Δ □ ° ◊ ▪ Good hydrophilicity Δ □ ° ◊ ▪ High electrochemical capacity ° Reversible ion intercalation/deintercalation ° Acts as a barrier for corrosion ▪ High mechanical strength ▪	Poor environmental stability Δ □ ° ◊ ▪
g‐C_3_N_4_	Photocatalytic activity in visible range □ Remarkable chemical stability Δ □ ° ◊ ▪ Tunable surface area and porosity Δ □ ° ◊ ▪ Narrow bandgap Δ □ °	High recombination rates of photogenerated electron and hole pairs □ Low electrical conductivity Δ □ ° Limited active sites Δ □ ° ◊ ▪
BP	Tunable bandgap Δ □ ° Excellent charge carrier mobility Δ □ ° Semiconductive properties Δ □ °	Poor chemical stability in water or under air Δ □ ° ◊ ▪

Applications: Δ (sensing), □ (catalysis), °(energy storage) ◊ (adsorption and filtration), ▪ (anticorrosion).

### 2.1 Enhanced COF Processability and Device Integration

COFs, typically synthesized as insoluble solid powders, often face limitations or drawbacks related to anisotropic growth and limited structural control, which hinder their processability and integration into devices. Hybridizing COFs with 2DMs in solution offers an effective solution by providing platforms that promote uniform growth and improve their processability. This approach minimizes irregularities and stacked COF structures that can impede mass transport and limit access to active sites. For instance, functionalizing GO with amino groups facilitates the anchoring of COF monomers, promoting uniform COF growth and enhancing structural stability.^[^ ^58^ ^]^ Similarly, MoS_2_ functionalized with (3‐aminopropyl)triethoxysilane (APTES) introduces amino groups that serve as nucleation sites for COF growth.^[^ ^59^ ^]^ Such hybridization strategies enable the creation of thin films that seamlessly integrate the structural rigidity of COFs with the flexibility of 2DMs, thereby enabling the efficient fabrication of thin films and advanced layered devices. This synergy is particularly advantageous for electronic applications, where uniformity and mechanical stability are critical. Thin films of graphene‐COF hybrids, for example, have demonstrated excellent mechanical stability and flawless integration into photodetector and sensing device architectures.^[^ ^60^, ^61^ ^]^ Additionally, scalable hybridization strategies, such as solvothermal and hydrothermal synthesis, enable cost‐effective large‐scale production of 2DM‐COF hybrids. These hybrids exhibit enhanced mechanical and chemical stability, ensuring compatibility with existing manufacturing workflows and expanding their potential for industrial settings.^[^ ^47^ ^]^

### 2.2 Enhanced Chemical Stability and Corrosion Resistance of COFs and 2DMs

The hybridization of COFs with 2DMs effectively addresses the inherent limitations in the chemical stability of COFs and the corrosion resistance of 2DMs, particularly under harsh environmental conditions. These enhancements are critical for applications such as energy storage, catalysis, and protective coatings. For instance, integrating Ti₃C₂Tx MXene with COF‐Lanzhou University 1 (COF‐LZU1) significantly improved its chemical stability and structural integrity during repeated charge–dischargecycles in corrosive electrolytes, achieving a coulombic efficiency exceeding 99% over 300 cycles. Similarly, GO‐COF hybrids demonstrated enhanced resistance to acidic degradation due to strong π–π interactions, maintaining high adsorption capacities even after multiple cycles in acidic solutions. In contrast, pristine COF‐LZU1 and Ti_3_C_2_T_x_ MXene showed a cycle life of less than 100 charge–discharge cycles, with significant capacity degradation under similar conditions.^[^ ^62^ ^]^

Moreover, the complementary properties of COFs and 2DMs enable outstanding performance in anticorrosion applications. While 2DMs like MoS₂ act as physical barriers, COFs provide a porous framework for encapsulating corrosion inhibitors. For example, MoS₂‐COF hybrids maintained superior resistance in corrosive environments by combining the mechanical strength of MoS₂ with the controlled release functionality of COFs. This dual‐action mechanism ensures prolonged protection and self‐healing capabilities, highlighting the advantages of hybrid systems over traditional anticorrosion materials.^[^ ^57^ ^]^

### 2.3 Mitigating Self‐Agglomeration of 2DMs

A persistent challenge with 2DMs, especially in liquid‐phase processing, is their tendency to self‐agglomerate, which significantly diminishes their effectiveness in various applications. The integration of COFs offers a robust solution to this issue by preventing the self‐agglomeration of 2DMs in liquid media. In rGO‐COF hybrids, the COF framework acts as a spacer, maintaining the dispersion of rGO sheets in both aqueous and organic solutions. This prevents the stacking of GO nanosheets, as demonstrated by Zhang et al. resulting in enhanced extraction efficiency of polycyclic aromatic hydrocarbons due to the synergistic effect of the materials.^[^ ^63^ ^]^ By mitigating aggregation, the individual sheets of 2DMs retain their high surface area and reactivity, ensuring full accessibility of their active sites for interactions with target species, tailored to specific applications. This property is essential for achieving reliable performance. For example, the hybridization of COFs and MXenes results in MXene‐COF heterostructures forming a framework suitable for numerous ion rectification and charge transfer operations, fully exposing active sites and greatly enhancing mass transfer efficiency.^[^ ^64^ ^]^

### 2.4 Improved Environmental Stability of 2DMs

Certain 2DMs, such as MXenes and BP, face challenges related to environmental stability. MXenes, while highly conductive, are prone to oxidation in aqueous and atmospheric conditions, severely limiting their long‐term functionality. Similarly, BP, known for its tunable bandgap and high carrier mobility, undergoes rapid degradation when exposed to ambient air and moisture, hindering its practical application. Hybridizing these materials with COFs significantly enhances their environmental stability. For instance, BP hybridized with triazine‐based COFs demonstrated significant improvements in resistance to oxidation and environmental degradation. The hybrid maintained its crystal structure and surface composition after prolonged exposure to air and moisture, as confirmed through X‐ray diffraction (XRD) and X‐ray photoelectron spectroscopy (XPS) analyses. This enhanced stability enabled superior performance in flame‐retardant applications.^[^ ^42^ ^]^ Similarly, Ti_3_C_2_T_x_ MXene‐COF hybrids showed enhanced resistance to oxidation and structural degradation. In lithium–sulfur batteries, the integration of a Ti_3_C_2_T_x_ MXene with a hydrazone‐linked COF prevented the restacking of MXene layers and reduced surface oxidation. This hybrid delivered excellent cycling stability, retaining 94% of its capacity after 100 cycles with a high sulfur loading of 5.6 mg cm^−2^, demonstrating superior performance compared to unprotected MXenes.^[^ ^65^ ^]^

### 2.5 Enhanced Surface Area and Increased Active Sites of 2DMs

As discussed in the previous section, 2DMs inherently possess high surface areas; however, this advantage is often compromised due to their tendency to self‐agglomerate. Hybridization with COFs not only mitigates self‐agglomeration but also enhances the effective surface area while introducing new functional groups derived from the COF structures. Although COFs generally exhibit higher surface areas than 2DMs, the resulting hybrid materials often achieve an intermediate surface area that balances performance across a range of applications. For instance, COFs can reach surface areas as high as 1300 m^2^ g^−1^, while rGO has a comparatively lower surface area of 88.1 m^2^ g^−1^. In rGO‐COF hybrids, the surface area reaches 119.8 m^2^ g^−1^, representing an intermediate value. Despite a reduction in surface area compared to pristine COFs, hybridization significantly increasing the number of exposed active sites on rGO. This enhancement leads to improved NO₂ gas sensing performance, with the hybrid achieving detection at concentrations as low as 250 ppb (2.7 times higher sensitivity than pristine rGO).^[^ ^61^ ^]^ Similarly, the surface areas of COF‐42, Ti_3_C_2_T_x_, and their hybrid Ti_3_C_2_T_x_‐COF are 1224, 20, and 565 m^2^ g^−1^, respectively. This hybridization creates a unique nanoarchitecture with hierarchical porous channels and abundant exposed active centers. As a result, the Ti₃C₂T_x_‐COF hybrid demonstrates exceptional hydrogen evolution reaction (HER) performance with a Tafel slope of only 50 mV dec^−1^, which is smaller than pristine COF‐42 (162 mV dec^−1^) and Ti₃C₂Tx (195 mV dec^−1^) as well as slightly inferior to that of Pt/C electrode.^[^ ^66^ ^]^

Notably, hybridization does not always yield an intermediate surface area. For example, the surface areas of COF 1,3,5‐triformylphloroglucinol (Tp)‐benzidine (BD), g‐C_3_N_4_, and their hybrid g‐C_3_N_4_‐COF Tp‐BD (10:1) amount to 351.97, 108.06, and 56.24 m^2^ g^−1^, respectively. Although the hybrid's surface area is lower than that those of the individual components, this reduction is offset by substantial gain in light absorption, efficient charge transfer, and improved photocatalytic activity, with a H_2_ production rate of 384.07 µmol·h^−1^, significantly surpassing the rates of pristine COF Tp‐BD (6.24 µmol·h^−1^), and pristine g‐C_3_N_2_ (1.35 µmol·h^−1^).^[^ ^41^ ^]^

### 2.6 Enhanced Electrical Conductivity and Charge Transfer of COFs

COFs typically exhibit low electrical conductivity, which limits their effectiveness in applications such as sensing, catalysis, and energy storage. Hybridization with conductive 2DMs like graphene, MXenes, or metallic‐phase MoS₂, significantly enhances charge transfer capabilities and overall performance. Graphene‐COF photodetectors exhibited an ultrahigh photoresponsivity of 3.2 × 10⁷ A W^−1^ at 473 nm and a response time of 1.14 ms, significantly surpassing the performance of pristine COF and graphene components. This enhancement arises from the improved charge transfer efficiency facilitated by the synergistic π–π interactions between the COF and graphene.^[^ ^60^ ^]^ Similarly, rGO‐COF hybrids demonstrated a remarkable enhancement in photocatalytic HER, achieving a H_2_ production rate 4.85 times higher than that of the pristine COF. This improvement is attributed to the covalent bonding between the COF and rGO, which facilitates efficient electron transfer and significantly enhances charge separation.^[^ ^67^ ^]^ Ti_3_C_2_T_x_ MXene‐COF hybrids demonstrated exceptional performance as electrodes for flexible supercapacitors, delivering a capacitance of 390 F g⁻¹ at 0.5 A g⁻¹, far exceeding the capabilities of pristine COFs (24 F g⁻¹) or Ti_3_C_2_T_x_ MXene (240 F g⁻¹) alone. This remarkable enhancement stems from the synergistic interaction between the highly porous structure of COFs and the excellent intrinsic conductivity of MXenes. The integration effectively alleviates the self‐restacking of MXene nanosheets while creating a well‐ordered framework that facilitates rapid electron transfer and ion migration.^[^ ^68^ ^]^

### 2.7 Improved Catalytic Activity of COFs and 2DMs

Hybridizing COFs with 2DMs has proven to be highly effective in enhancing catalytic performance by addressing the intrinsic limitations of each material. While COFs offer tunable catalytic properties, they suffer from poor charge transport and rapid electron–hole recombination. Similarly, certain 2DMs, such as MXenes, exhibit excellent electrical conductivity, others, like rGO, MoS_2_, and g‐C_3_N_4_ face challenges with rapid electron–hole recombination, significantly limiting their catalytic efficiency. Moreover, many 2DMs lack the structured porosity and high density of active sites that are COFs inherently possess. Hybridizing these materials creates powerful synergies, significantly boosting catalytic efficiency through optimal energy level alignment, reduced electron–hole recombination, enhanced charge transfer, and improved accessibility to active sites. For example, a MoS_2_‐COF heterostructure engineered for photoelectrochemical (PEC) water oxidation exhibited a photocurrent density of 2.5 µA cm⁻^2^ at 1.23 V versus the reversible hydrogen electrode (RHE), representing twice the performance of photoanodes made from pristine COFs. This enhancement is due to the optimal energy band alignment between MoS_2_ and the COF, which promotes efficient charge separation and transfer, thereby minimizing electron–hole recombination.^[^ ^69^ ^]^ In another example, Ti_3_C_2_T_x_ MXene‐COF hybrids exhibited exceptional performance in the electrocatalytic reduction of CO_2_ to CO. The hybrid achieved a Faradaic efficiency of 97.28% for CO_2_‐to‐CO conversion at −0.6 V versus RHE, far surpassing the efficiency of either pristine COFs or Ti_3_C_2_T_x_ MXene alone. The improved performance is due to the synergistic combination of MXene's high electrical conductivity and COF's abundant active sites and structured porosity, enabling more efficient charge transfer and reactant diffusion.^[^ ^70^ ^]^

### 2.8 Enhanced Mechanical Stability of COFs

The mechanical properties of 2DM‐COF hybrids are significantly enhanced through hybridization, leveraging the complementary strengths of their components. For instance, Ti_3_C_2_T_x_ MXene‐COF hybrids combine the mechanical robustness of MXenes with the structural flexibility and porosity of COFs, resulting in composites that are highly resistant to mechanical stress and deformation. This makes them ideal for robust coatings and structural applications. A notable example is the development of NH₂‐functionalized Ti_3_C_2_T_x_ MXene‐COF hybrids in epoxy nanocomposite coatings, which demonstrated an 80% increase in tensile strength, rising from 26.60 MPa in neat epoxy to 46.60 MPa in the hybrid system.^[^ ^71^ ^]^

### 2.9 Regulated Wettability and Improved Ion Mobility of COFs

Hybridizing with hydrophilic 2DMs like MXenes, GRM, or functionalized MoS_2_ significantly enhances the wettability of COFs, leading to superior performance in energy storage and catalysis. These hybrids exhibit faster ion transport and better electrolyte accessibility, making them suitable for supercapacitors and batteries. For example, a graphene‐COF hybrid demonstrated significantly improved wettability and electrochemical performance. The hybrid achieved a specific capacitance of 321 F g⁻¹, compared to only 110 F g⁻¹ for pristine COFs and 203 F g⁻¹ for rGO. This enhancement is attributed not only to the COF's porous structure, which prevents the restacking of graphene nanosheets, but also to the improved wettability of the hybrid. The increased wettability ensures better interaction with the electrolyte, facilitating ion diffusion and charge transfer, which are critical for achieving high‐performance energy storage systems.^[^ ^72^ ^]^

## 3 Hybridization Strategies

**Figure 3:**
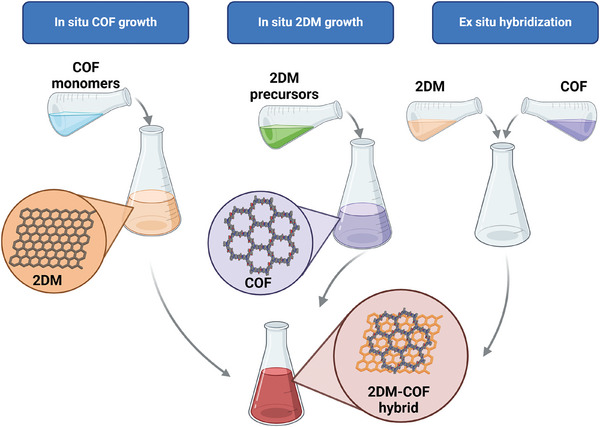
In situ solvothermal growing of COF on 2DM surface; in situ hydrothermal synthesis of 2DM on COF surface and ex situ hybridization of 2DM and COF dispersion.

**Figure 4:**
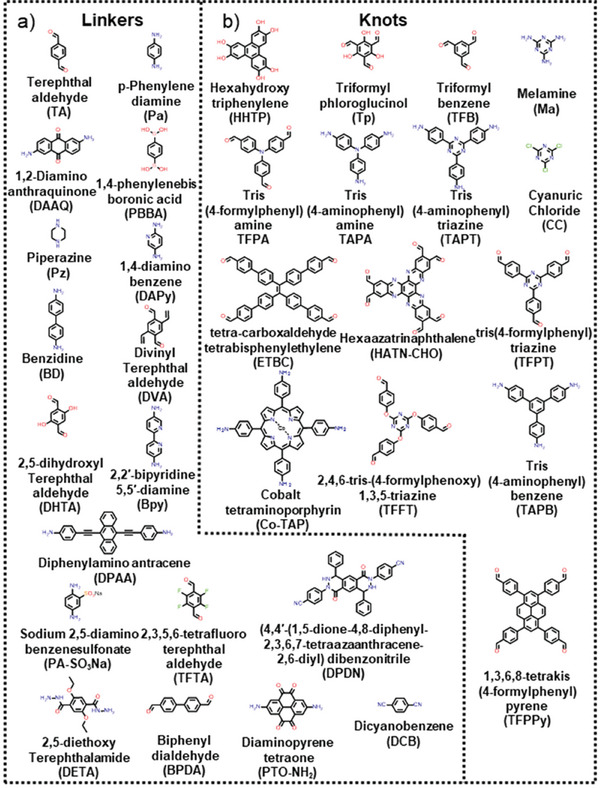
List of COF monomers described in this review is divided as a) linkers and b) knots.

Hybrid structures comprising 2DMs and COFs can be generated by means of three distinct pathways as illustrated in **Figure** **3**: i) solvothermal in situ synthesis of COF on the surface of 2DM; ii) hydrothermal in situ synthesis of 2DM on the surface of COF, and iii) ex situ synthesis of both components followed by their hybridization via blending. The selection of the hybridization strategy is determined by the chosen 2DM, in view of its reactivity and affinity for the COFs of interest. **Figure** **4** displays the chemical structures of all the COF monomers that have been employed for the fabrication of 2DM‐COF hybrids, to the best of our knowledge, to date. Noteworthy, all these monomers lead to the formation of 2D COFs. This is due to the inherent compatibility between 2DMs and 2D COFs, as their planar structures enable close interfacial contact and promote efficient charge transfer.

### 3.1 Hybridization Strategies and their Influence on Physicochemical Properties and Performance

The choice of synthesis method plays a critical role in determining the physicochemical properties of 2DM‐COF hybrids. These methods influence their structural characteristics, stability, and functional performance (**Table** **2**). Three primary hybridization strategies, each with unique advantages and limitations, govern the design and application of these hybrids.

**Table 2:** Comparative analysis of synthesis strategies for 2DM‐COF hybrids in various applications.

Application	Solvothermal in situ synthesis of COF on the surface of 2DM	Hydrothermal in situ synthesis of 2DM on the surface of COF	Ex situ synthesis of both components followed by hybridization via blending
Chemical sensing	Enhanced sensitivity and selectivity toward analytes due to high active site exposure	Faster response times and lower detection limits due to enhanced wettability and conductivity	Moderate performance due to less optimized energy level alignment and interfacial stability
Catalysis	Efficient charge transfer reduces electron–hole recombination and enhances stability	The strong interfacial bonding and hydrophilic nature promote efficient reactant diffusion and enhanced activity	Useful for exploratory studies, though catalytic efficiency is often lower due to weaker charge transfer pathways
Energy storage	Uniform porosity and enhanced conductivity enable higher electrochemical performance and long‐term cycling stability	Reduced interfacial resistance and better ion transport significantly boost capacitance and rate performance	Suitable for low‐cost devices, but cycling stability and rate capability are typically inferior to in situ hybrids
Adsorption and filtration	High porosity facilitates greater adsorption capacity and selectivity for gases or organic pollutants	Controlled 2DM deposition increases filtration efficiency, particularly for water treatment and gas separation	Effective for bulk adsorbents, particularly in large‐scale water or air purification systems
Anticorrosion	Strong interfacial bonding between the COF and 2DM creates robust protective barriers, significantly increasing resistance to corrosive agents	The uniform 2DM layers provide excellent physical and chemical barriers, extending the durability of coatings under aggressive environmental conditions	Provides basic protection but lacks the robustness of in situ hybrids, making them less durable in highly corrosive environments

#### 3.1.1. Solvothermal In Situ Synthesis of COF on the Surface of 2DM

This strategy involves the growth of COFs directly on the surface of 2DMs under solvothermal conditions, yielding close integration of the two materials and ensures robust interfacial interactions. The resulting hybrids exhibit a range of enhanced properties.

Uniform growth of COFs on the 2DM surface leads to well‐ordered hybrid structures with optimized porosity and consistent layer stacking. Strong covalent or p‐p interactions significantly improve chemical and mechanical stability, enabling the hybrids to withstand harsh conditions. Furthermore, the direct synthesis process promotes excellent energy level alignment, resulting in superior charge transfer efficiency. In terms of applications, this hybridization method yields outstanding results across various domains. In chemical sensing, the highly ordered structure maximizes active site exposure, enhancing both sensitivity and selectivity toward analytes. The hybrid material consisting of a COF, formed by 1,3,5‐tris‐(4‐aminophenyl)triazine (TPAT) and 2,5‐dihydroxyl‐terephthalaldehyde (DHTA), amine‐functionalized GO (GO‐NH_2_), and metallic gold nanoparticles (Au NPs) (Au@COF/GO‐NH_2_) employed as electrochemical aptasensor exhibits significantly improved sensitivity and selectivity for analytes including chloramphenicol (CAP) when compared to pristine GO‐NH_2_ and TAPT‐DHTA COF.^[^ ^73^ ^]^ Sensitivity expressed as ΔRct, reflects the variation in charge transfer resistance before and after analyte interaction. GO‐NH_2_ and TAPT‐DHTA COF show smaller ΔRct (0.109 and 0.318 kohm, respectively) due to their limited conductivity and active site accessibility. In contrast, the hybrid Au@COF/GO‐NH_2_ material yields a substantial ΔRct increase of 0.475 kohm. This notable improvement is attributed not only to the inclusion of Au nanoparticles, which enhance conductivity, but also to the higher active site availability in the hybrid porous material.^[^ ^73^ ^]^ For catalysis, the efficient charge transfer reduces electron–hole recombination and enhances stability, making these hybrids effective in photocatalysis and electrocatalysis. In the case of the g‐C_3_N_4_‐COF (10:1) hybrid, a 2D g‐C_3_N_4_‐1D COF heterojunction structure is constructed, enabling a high degree of visible light absorption and effective charge separation.^[^ ^41^ ^]^ The well‐matched bandgaps between COF Tp‐BD and g‐C_3_N_4_ facilitate rapid electron transfer, significantly boosting photocatalytic hydrogen production rates to 12.8 mmol g^−1^h^−1^, which is 62 and 284 times higher than the individual COF and g‐C_3_N_4_, respectively.^[^ ^41^ ^]^ The uniform porosity and enhanced conductivity also translate to superior energy storage capabilities, offering higher electrochemical performance and long‐term cycling stability. In the case of the MXene‐COF hybrid, the covalent assembly of ultrathin COF‐LZU1 on Ti_3_C_2_T_x_ MXene nanosheets creates a robust 2D heterostructure with hierarchical porosity and excellent charge transport properties.^[^ ^62^ ^]^ The structure of the hybrid promotes fast lithium‐ion diffusion through uniform nanochannels and prevents dendritic lithium growth by ensuring homogeneous nucleation. These features result in exceptional lithium storage performance, including stable cycling at high current densities (up to 20 mA cm^−2^) and a 300% increase in cycle life compared to bare copper electrodes.^[^ ^62^ ^]^ In adsorption and filtration, high porosity facilitates greater adsorption capacity and selectivity for gases or organic pollutants. The rGO‐COF Tp‐p‐phenylen diamine (Pa) nanocomposite, developed through solvothermal method, exemplifies this by combining the capacitive properties of rGO with the redox‐active COF Tp‐Pa.^[^ ^74^ ^]^ This hybrid material achieves a Pb^2+^ adsorption capacity of 137.8 mg g^−1^ from a 100 mg L^−1^ Pb(NO_3_)_2_ solution and an impressive removal efficiency of 99.6% in the presence of competing ions such as Na^+^, K^+^, Mg^2+^, and Ca^2+^. The strong interfacial bonding between COF Tp‐Pa and rGO provides abundant nitrogen and oxygen sites for selective Pb^2+^ binding, while the porous structure enhances ion accessibility. The integration of COF Tp‐Pa and rGO ensures high conductivity, facilitating fast ion transfer and accelerating adsorption processes.^[^ ^74^ ^]^ Additionally, the robust interfacial bonding provides superior anticorrosion properties, creating highly durable protective barriers against corrosive agents. As an example, a hybrid material composed of amino‐functionalized Ti_3_C_2_T_x_ MXene and COF melamine (Ma) and terephthalaldehyde (TA) loaded with zinc ions and glutamate, ensured an excellent corrosion protection.^[^ ^75^ ^]^ The hybrid achieves an 88% corrosion inhibition efficiency over 96 h in a saline environment, compared to significantly lower performance in traditional coatings. Furthermore, when incorporated into an epoxy matrix, the hybrid material improves active corrosion resistance by over 200% compared to unmodified epoxy coatings, providing long‐term protection under harsh conditions, such as in 3.5 wt% saline solutions.^[^ ^75^ ^]^

#### 3.1.2. Hydrothermal In Situ Synthesis of 2DM on the Surface of COF

In this approach, 2DMs (e.g., MoS_2_) are synthesized hydrothermally on pre‐formed COFs. By ensuring nanoscale integration, this method yields hybrids with robust interfacial interactions. The hydrophilic nature of COFs enhances wettability, facilitating better electrolyte interaction and ion transport. Furthermore, precise control over the morphology of 2DMs ensures consistent hybrid performance. These properties enable significant advancements in practical applications. Enhanced wettability and conductivity boost chemical sensing performance, enabling faster response times and lower detection limits. In the case of the MoS_2_‐COF Tp‐Pa composite, wettability is improved by the COF's porous structure and functional groups, which facilitate effective interaction with aqueous solutions and enhance the adsorption of analytes like Ni^2+^.^[^ ^76^ ^]^ Conductivity is significantly increased by the incorporation of MoS_2_, forming a 2D–2D heterojunction with COF that promotes efficient charge separation and transfer. Furthermore, the alignment of energy levels between MoS_2_ and COF Tp‐Pa enhances photochemical vapor generation (PVG) efficiency by reducing electron–hole recombination, enabling highly sensitive detection of trace heavy metals with superior anti‐interference capabilities.^[^ ^76^ ^]^ For catalysis, the strong interfacial bonding and hydrophilic nature promote efficient reactant diffusion and enhanced activity, particularly under aqueous conditions. In the case of the WS_2_‐covalent triazine framework (CTF‐1‐G) heterojunction, the integration of hyper‐crosslinked CTF‐1‐G and WS_2_ nanoflowers creates a Z‐type charge transfer pathway, facilitating efficient photogenerated carrier separation.^[^ ^44^ ^]^ The hydrophilic and porous structure of CTF‐1‐G enhances light absorption and reactant accessibility, while the strong interfacial bonding between the two components ensures stable and effective charge transfer. As a result, this hybrid system achieves superior catalytic performance for environmental remediation and hydrogen generation.^[^ ^44^ ^]^ Despite its great potential, to the best of our knowledge, this synthetic strategy has not been applied to hybrids with applications in energy storage, adsorption and filtration and anticorrosion. Nevertheless, we anticipate following synergies to appear: in energy storage, reduced interfacial resistance and improved ion transport significantly boost both capacitance and rate performance. The controlled deposition of 2DMs also improves adsorption and filtration efficiency, particularly for water treatment and gas separation. Furthermore, the uniform 2DM layers serve as excellent physical and chemical barriers, extending the durability of anticorrosion coatings under aggressive environments.

#### 3.1.3. Ex Situ Synthesis of Both Components Followed by Hybridization via Blending

In this method, pre‐synthesized COFs and 2DMs are physically blended to create the hybrids. This straightforward approach often involves mechanical mixing or solvent‐assisted dispersion. While it offers versatility and scalability, it is characterized by weaker interfacial bonding compared to in situ methods. Aggregation of 2DMs may occur, leading to reduced effective surface area and diminished charge transfer efficiency. Nonetheless, this method is cost‐effective and well‐suited for large‐scale production. The application performance of this class of hybrids tends to be moderate. In chemical sensing, weaker interfacial interactions and energy level alignment result in less optimized sensitivity and selectivity. The hybrid of amino‐functionalized multi‐walled carbon nanotubes (MWCNTs)‐COF (NH_2_‐MWCNT‐COF) and MoS_2_, used in electrochemical sensors for sulfamerazine detection, highlights the advantages and limitations of the ex situ fabrication method involving subsequent deposition.^[^ ^39^ ^]^ In this approach, NH_2_‐MWCNT‐COF and MoS_2_ are sequentially deposited onto the electrode surface, ensuring a controlled layering process that integrates the high conductivity and surface area NH_2_‐MWCNT‐COF with the electron transfer capabilities of MoS_2_. Although the use of this method prevents the random aggregation commonly associated with physical blending, challenges remain in maintaining optimal interface interactions and achieving uniform coverage across the layers. Nevertheless, the process is cost‐effective and scalable, making it a practical choice for sensor fabrication.^[^ ^39^ ^]^ For catalysis, exploratory applications are feasible, though the catalytic efficiency may be hindered by suboptimal charge transfer pathways. In the case of COF Nanyang Technological University (NTU) Tp‐Pa integrated with NH_2_‐Ti_3_C_2_T_x_ MXene, this challenge is addressed through a covalent coupling strategy that forms a 2D/2D heterojunction.^[^ ^40^ ^]^ This hybrid structure significantly enhances photocatalytic hydrogen production under visible light, achieving rates 12.6 times higher than pure NTU‐Tp‐Pa. The improvement stems from efficient charge separation, reduced recombination, and optimized interfacial interactions facilitated by the covalent bonding.^[^ ^40^ ^]^ In energy storage, this class of hybrids is suitable for low‐cost devices, but cycling stability and rate capability are fall short of in situ hybrids. For instance, the Ti_3_C_2_T_x_ MXene‐COF‐LZU1 composite framework enhances lithium‐sulfur battery performance by improving lithium nucleation and deposition through the lithiophilic COF‐LZU1 particles.^[^ ^77^ ^]^ The hybrid achieves a Coulombic efficiency of 97.6% at a current density of 0.5 mA·cm^−2^, significantly higher than the 78.7% of pristine Ti_3_C_2_T_x_ MXene. Additionally, the nucleation overpotential for lithium plating on the hybrid is reduced to 73.5 mV, compared to 130.5 mV for pristine Ti_3_C_2_T_x_ MXene, indicating more uniform lithium deposition. However, after 300 cycles at 1 C, the hybrid retains 73.8% of its initial capacity (dropping from 1152.8 to 850.7 mAh·g^−1^, whereas the pristine system retains only 19.3% (decreasing from 1096.8 to 211.4 mAh·g^−1^). These numbers reflect the improvements but also the limitations of the ex situ method in achieving in situ‐level performance.^[^ ^77^ ^]^ The method finds utility in bulk adsorption and filtration applications, such as large‐scale water or air purification. For example, partially reduced GO (prGO) laminates intercalated with 2D TFB‐Pa COFs have been developed into robust nanofiltration membranes with exceptional performance.^[^ ^78^ ^]^ The intercalation of COF nanosheets into prGO prevents restacking and increases interlayer spacing, resulting in a 27‐fold enhancement in water permeance compared to pristine prGO membranes, reaching up to 194.0 L·m^−2^·h^−1^·bar^−1^. This improvement is achieved without compromising selectivity, maintaining rejection rates above 98% for dyes such as methylene blue, rhodamine B, and acid orange.^[^ ^78^ ^]^ However, in anticorrosion applications, these hybrids provide only basic protection, lacking the robustness needed for highly corrosive environments. To the best of our knowledge, this synthetic strategy has not been applied to hybrids with applications in anticorrosion.

### 3.2 Hybridization of Graphene and Graphene‐Related Materials with COFs

The preparation of the first graphene–COF hybrid material was achieved by the in situ solvothermal growth of a boronic ester‐linked COF in the presence of graphene nanosheets.^[^ ^36^ ^]^ Dichtel et al. reported the preparation of graphene‐COF by dissolving the 1,4‐phenylenebis boronic acid (PBBA) and 2,3,6,7,10,11‐hexahydroxytriphenylene (HHTP) in a mixture of mesitylene:dioxane (1:1 v/v) at 90 °C (i.e., COF‐5) in the presence of single‐layer graphene (SLG) supported on copper film.

The solvothermal growth of COF on the surface of graphene sheets was driven by p‐p interactions between the extended conjugated structure of graphene and the benzene rings of the COF monomers. However, this approach is limited to COF monomers with extended p‐conjugation that can maximize interactions with the graphene layer. To facilitate COF growth on a GRM surface, a potential strategy involves the surface functionalization of the GRM with small organic molecules that can covalently bridge the GRM sheets and the COF. Among all graphene derivatives, GO is particularly suitable as it can be easily functionalized with primary amines through the ring opening of epoxy groups, and the amidation of carboxylic moieties. Imine‐based COF growth on GO uses functionalized GO as nucleation sites, enhancing bonding and structural stability, though it requires long reaction times and careful control to avoid aggregation. Key challenges across these methods include achieving uniform COF growth while preserving graphene's electronic properties. Li et al. reported the preparation of a GO‐imine‐based COF hybrid by incorporating a GO dispersion in dioxane to the COF reaction mixture.^[^ ^58^ ^]^ In this process, one amino group of 2,6‐diaminoanthraquinone (DAAQ), used as diamine linker for the COF synthesis, covalently reacts with the epoxy and carboxyl groups on GO surface. The second amino group extends outward, serving as a nucleation site for COF growth. Similarly, Pan et al. developed a synthetic protocol for the imine‐based COF hybridization with GO nanosheets.^[^ ^79^ ^]^ While GO nanosheets were dispersed in water, the COF monomers (Tp and DAAQ) were mixed with *p*‐toluenesulfonic acid. In the resulting slurry dispersion, the protonated monomers were adsorbed onto the GO sheets surface through hydrogen bonds. Consequently, by raising the temperature to 120 °C for 3 days, the imine‐based COF exhibited a rapid growth along the graphene skeleton, guided by the previously formed hydrogen bonds and crosslinking the COF surface to the GO surface through amide and amine bonds.

### 3.3 Preparation of MoS_2_‐COF Hybrids

**Figure 5:**
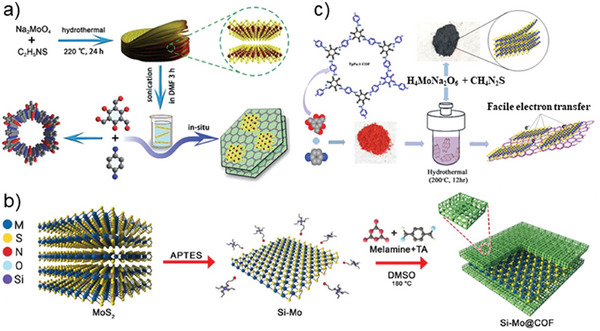
a) MoS_2_‐COF hybridization through solvothermal COF growth; Reproduced with permission.^[^ ^80^ ^]^ Copyright 2019, Royal Society of Chemistry; b) MoS_2_‐COF hybridization through APTES crosslinker approach, Reproduced with permission.^[^ ^57^ ^]^ Copyright 2022, American Chemical Society; and c) MoS_2_‐COF hybridization through hydrothermal MoS_2_ growth. Reproduced with permission.^[^ ^81^ ^]^ Copyright 2020, American Chemical Society.

Zhang et al. reported for the first time a synthetic protocol for the hybridization of imine‐based COF and MoS_2_ through a solvothermal approach, where COF sheets were grown in situ in the presence of MoS_2_ flakes (**Figure** **5**a). This solvothermal approach is straightforward but requires solvent optimization (e.g., using DMF for MoS₂ dispersion). The authors modified a well‐known COF Tp‐Pa synthesis protocol by substituting the solvent mixture (dioxane/mesitylene) with dimethylformamide (DMF). This change allowed them to use the MoS_2_ dispersion in DMF, obtained after exfoliation, directly for the solvothermal MoS_2_‐COF hybridization.^[^ ^80^ ^]^ To enhance the stability of MoS_2_‐COF hybrids through covalent crosslinking, the MoS_2_ surface is often preliminarily functionalized with organic small molecules.

However, unlike GO, MoS_2_ surface does not achieve stable functionalization with diamino molecules. The literature reported functionalization of MoS₂ is restricted to siloxane molecules, such as APTES, which enhances covalent bonding for imine‐based COF growth but limits the versatility of the functionalization process compared to other materials. The siloxane moiety forms a strong covalent bond with the MoS_2_ surface, allowing the amino group to protrude and initiate the growth of the imine‐based COF. For instance, Arjmand et al. silanized the MoS_2_ surfaces with APTES, followed by a solvothermal growth of an imine‐based COF using Ma and TA (Figure 5b).^[^ ^57^ ^]^ The remarkable chemical stability of imine and b‐keto enamine COFs makes them suitable for the hydrothermal growth of MoS_2_ nanoplates on the COF surface. Hydrothermal growth of MoS₂ nanoplates on COF templates offers an alternative method, though it requires high temperatures and precise control to ensure uniform MoS₂ deposition. General challenges for the synthesis of MoS₂‐COF hybrids include achieving stable functionalization and consistent MoS₂‐COF integration without compromising stability. Within this regard, Zhang et al. reported the first MoS_2_‐COF hybrid obtained through a facile hydrothermal growth of MoS_2_ layers on a COF template formed by Tp and Pa (COF Tp‐Pa).^[^ ^81^ ^]^ The reported protocol envisages the addition of thiourea and sodium molybdate (Na_2_MoO_4_)×2H_2_O to an aqueous dispersion of COF Tp‐Pa powder, keeping the reaction mixture for 12 h at 200 °C (Figure 5c).

### 3.4 Generation of MXene‐COF Hybrids

**Figure 6:**
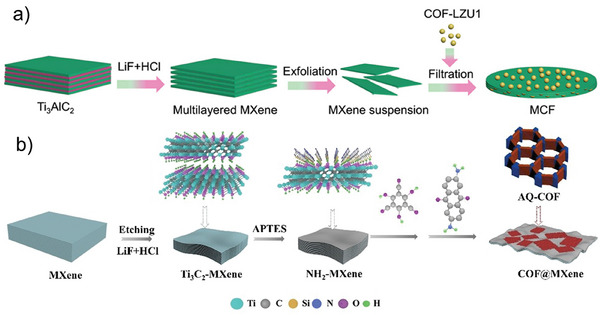
a) MXene‐COF hybridization through mixing approach, Reproduced with permission.^[^ ^77^ ^]^ Copyright 2021, Springer Nature; and b) MXene‐COF hybridization through APTES crosslinker approach Reproduced with permission.^[^ ^82^ ^]^ Copyright 2022, Wiley‐VCH.

The ex situ synthesis method for hybridizing COFs and MXenes involves assembling MXene nanosheets with exfoliated 2D COF sheets by simply blending the suspensions of the two materials. The cohesion between COF and MXene flakes is primarily maintained through noncovalent interactions, especially electrostatic interactions. For example, Feng et al. reported the preparation of a MXene‐COF hybrid by sonicating together a suspension of COF‐LZU1 and Ti_3_C_2_T_x_ MXene solution for 10 min, followed by vacuum filtration and drying at 60 °C under vacuum conditions (**Figure** **6**a).^[^ ^77^ ^]^ Similarly, Dong et al. reported the preparation of a MXene‐COF hybrid material by combining Ti_3_C_2_T_x_ MXene and an anthraquinone‐based COF (COF DAAQ‐Tp) which was pre‐protonated to enhance the electrostatic interactions between the positively charged COF layers and the partially negatively charged MXene nanosheets.^[^ ^68^ ^]^ Ex situ synthesis method is simple but lacks strong covalent bonds, which can affect stability. Using an in situ ionothermal approach, Yang et al. hybridized CTF, obtained through trimerization of 1,4‐dicyanobenzene (DCB), with Ti_3_C_2_ MXene nanosheets.^[^ ^65^ ^]^The in situ CTF polymerization on the MXene surface was performed in a vacuum tube at 400 °C in the presence of molten zinc chloride salt (ZnCl_2_) that worked as both solvent and catalyst. This process resulted in the formation of a stable interface, characterized by covalent Ti‐N interactions between the MXene nanosheet and CTF layer, facilitating the interfacial electron transfer and inducing atomic charge polarization between the CTF and MXene. This approach avoids the use of environmentally harmful solvents and catalysts in large quantities and also reduces reaction times compared to conventional synthetic protocols.^[^ ^15^ ^]^ However, this method requires high temperatures (400°C) and molten salts as solvents.

The covalent crosslinking of COFs on MXene nanosheets is the most studied method of hybridization. Functionalizing MXene with crosslinkers for covalent COF growth enhances the uniformity and stability but depends on the functional groups from MXene etching, presenting challenges in optimization. The overall challenges in hybridizing COFs with MXenes involve balancing simplicity, stability, and the complexity of synthesis methods. This process involves two main steps: 1) functionalization of the MXene surface with a crosslinker to initiate COF growth; 2) solvothermal growth of the COF on the MXene surface. The functionalization step is determined by the nature of functional groups on the MXenes surface after wet‐etching (oxygen (–O), fluorine (–F), hydroxyl (–OH), and chloride (–Cl)).^[^ ^83^ ^]^ For instance, Wang et al. reported the hybridization of Ti_3_C_2_ MXene with enamine‐based COF DAAQ‐Tp by using APTES as a crosslinker (Figure 6b).^[^ ^82^ ^]^ The ‐NH_2_ terminal groups of the APTES moiety act as nucleation sites for the Schiff‐base formation through condensation with the C = O groups from the COF aldehydic monomer. This enables the uniform growth of the COF nanosheets on the surface of Ti_3_C_2_ MXenes.

### 3.5 Strategies Toward g‐C_3_N_4_‐COF Hybrids

**Figure 7:**
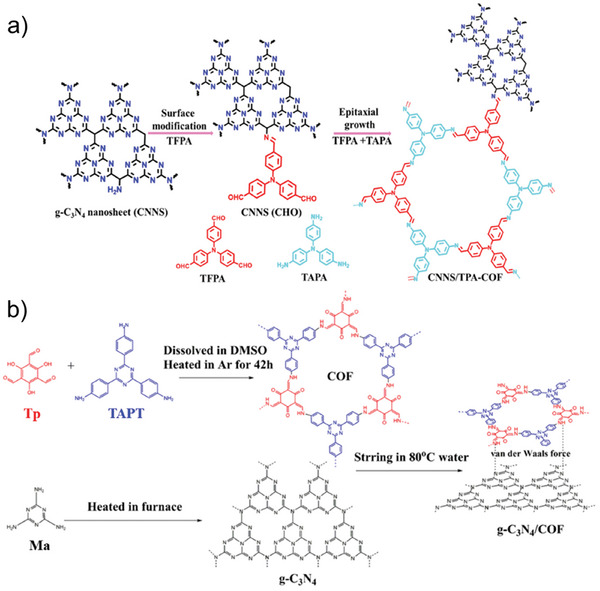
a) Schematic illustration of the in situ preparation of the CNNS/TPA‐COF 2D/2D heterojunction photocatalysts, Reproduced with permission.^[^ ^84^ ^]^ Copyright 2023, Royal Society of Chemistry; and b) schematic illustration for the ex situ preparation of g‐C_3_N_4_/COF van der Waals heterojunction, Reproduced with permission.^[^ ^85^ ^]^ Copyright 2022, Elsevier Science & Technology Journals.

The g‐C_3_N_4_‐COF hybridization has been investigated primarily with either in situ COF growth on the 2DM surface or ex situ physical mixing. Xu et al. designed and synthesized a covalently linked g‐C_3_N_4_‐COF hybrid material by in situ growing of COF on g‐C_3_N_4_ nanosheets.^[^ ^84^ ^]^ The presence of unreacted primary amino groups makes it possible to initialize the imine‐base COF growth directly on the g‐C_3_N_4_ surface by using the aldehyde monomer as a crosslinker. The g‐C_3_N_4_ nanosheets (CNNSs) were preliminarily functionalized with the tris‐aldehyde monomer, tris(4‐formylphenyl)amine (TFPA), forming CNNS with pending formyl groups (CNNS‐CHO) (**Figure** **7**a). Subsequently, COF was grown on CNNS surface through a solvothermal method by adding CNNS‐CHO to the COF reaction mixture which included TFPA and tris(4‐aminophenyl)amine (TAPA). The prepared CNNS/TPA‐COF showed a remarkable crystallinity, high porosity, and a wide visible‐light absorption, making it an ideal material for photocatalytic applications.^[^ ^84^ ^]^ This method provides strong covalent bonding, resulting in high crystallinity, porosity, and enhanced light absorption, ideal for photocatalysis. However, the process is complex and requires careful functionalization.

Ye et al. hybridized defective g‐C_3_N_4_ nanosheets with an enamine‐based COF through an ex situ self‐assembly approach by mixing pre‐synthetized COF and g‐C_3_N_4_ (Figure 7b).^[^ ^85^ ^]^ The defective g‐C_3_N_4_ was obtained by Ma thermal annealing in a tubular oven under 90% Ar and 10% H_2_ for 4 h at 550 °C. The hydrogen‐enriched atmosphere created additional nitrogen vacancies and facilitated the exfoliation of g‐C_3_N_4_ into ultrathin nanosheets. The COF was obtained by condensation reaction between Tp and tris(4‐aminophenyl)triazine (TAPT) in dimethyl sulfoxide (DMSO) without the use of a catalyst. The as‐prepared defective g‐C_3_N_4_ and COF Tp‐ TAPT were dispersed in water and stirred at 80 °C in an open flask until the solvent was totally evaporated. The hybridization between g‐C_3_N_4_ and COF Tp‐ TAPT is driven by p‐p interactions forming a van der Waals heterojunction. Ex situ physical mixing is simpler, where pre‐synthesized COFs and defective g‐C_3_N_4_ nanosheets are forming van der Waals heterojunctions via π‐π interactions. However, the lack of the strong covalent bonds seen in in situ methods, can potentially affect the hybrid stability. The main challenges in synthesizing g‐C₃N₄‐COF hybrids involve balancing complexity and stability. In situ methods provide strong covalent bonds, enhancing crystallinity and light absorption, but are complex and require precise functionalization. Ex situ methods are simpler but rely on weaker π‐π interactions, which may compromise stability.

### 3.6 Synthesis of BP‐COF Hybrids

Aly et al. reported for the first time the synthesis of a BN‐COF hybrid via a one‐pot interfacial polymerization. Melamine is poly‐condensed with terephthaldehyde in the presence of BN leading to the formation of a series of highly cross‐linked microporous hierarchical porous amine networks.^[^ ^86^ ^]^

Similarly, a triazine based organic framework was grown onto the surface of 2D BP by in situ polymerization, resulting in the formation of the first BP‐COF hybrid.^[^ ^42^ ^]^ The one‐pot interfacial polymerization method offers the advantage of simplicity, as it combines all components in a single step, promoting efficient covalent bonding and leading to highly cross‐linked, microporous structures. However, controlling the uniformity and morphology of the final hybrid can be challenging. Other BP‐COF hybrids have been synthesized through ex situ methods, either by mixing the pre‐formed components followed by a hydrothermal process or simply by using sonication and stirring.^[^ ^87^, ^88^ ^]^ These ex situ methods are simpler and faster but may lack the strong covalent bonding and interface stability of in situ approaches. This can lead to reduced structural integrity and performance in some applications.

## 4 Applications

In the following section, we introduce the most remarkable applications of 2DM‐COF hybrids in different fields, including sensing, catalysis, energy storage, adsorption and filtration, and others applications including anticorrosion, and flame retardant materials.

### 4.1 Sensing

Sensors are devices that can detect and quantify changes in the environment providing an electrical or optical output. Sensors can be sub‐divided based on the type of signal transduction method.

#### 4.1.1 Electrical Sensors

Electrical sensors offer advantages such as high sensitivity, rapid response times, and the ability to provide continuous real‐time data. Zhao et al. devised an innovative solution to the limited carrier mobility of COFs by creating a hybrid heterostructure combining a highly porous COF and rGO (yielding rGO‐COF).^[^ ^61^ ^]^ 1,3,5‐triformylbenzene (TFB) and TAPT were chosen as COF monomers due to their large specific surface area and high thermal stability. The resulting heterostructure was tested in chemiresistors for detecting hazardous gases, allowing the detection of NO_2_ gas at concentrations from 250 ppb to 5 ppm (**Table** **3**). The rGO‐COF heterostructure‐based sensor demonstrated a 2.7‐fold increase in sensitivity to NO_2_ and significantly reduced the response time to 32 seconds compared to pristine rGO (234 seconds). The integration of rGO and COF increased the number of adsorption sites and binding energies for NO_2_, leading to rapid adsorption and diffusion of gas molecules, thereby enhancing the gas sensing performance.

**Table 3:** Key performance indicators of the sensing performance of 2DM‐COF hybrids.

COF	2DM	Analyte	Sensor type	Detection range	Sensitivity	Limit of detection	Response time	Selectivity	Refs.
TFB‐Pa	Graphene	Photodetector	Photoresistor	–	Responsitivty: 0.5 ± 0.3 A W^−1^	–	1.20 s	–	[89]
ETBC–TAPT	Graphene	Photodetector	Chemiresistor	–	Responsitivty: 3.2 × 10^7^ A W^−1^ at 473 nm	–	1.14 × 10^−3^ s	–	[60]
TFB‐TAPT	rGO	NO_2_	Chemiresistor	250 ppb to 5 ppm (1.02 × 10^−5^ to 2.05 × 10^−4^ M))	ΔR/R_0_ = −36.1%	–	32 s	–	[61]
NH_2_‐MWCNT@COF	MoS_2_	Sulfamerazine	Electrochemical	3.0 × 10^−7^ to 2.0 × 10^−4^ M	–	1.1 × 10^−7^ M	–	Yes (sulfadiazine, sulfacetamide, and sulfathiazole))	[39]
DHTA‐TAPT	GO‐NH_2_	Chloramphenicol	Electrochemical	1.55 × 10^−13^ to 3.09 × 10^−9^ M	0.72 (slope of linear regression)	4.99 × 10^−14^ M	–	Yes (oxytetracycline, ciprofloxacin, neomycin sulfate, and penicillin	[73]
Tp‐Pa‐NO_2_	Ti_3_C_2_T_x_‐MXene	CYFRA21‐1	Electrochemical	0.5 to 1.0 × 10^4^ pg mL^−1^	–	0.1 pg mL^−1^	–	Yes (BSA, uric acid, carcinoembryonic antigen, dopamine, and ascorbic acid	[90]
Tp‐Pa	MoS_2_	Nickel	Optical	0.1 to 10 µg L^−1^ (1.70 × 10^−9^ to 1.70 × 10^−7^ M)	–	5.11 × 10^−10^ M	‐	Yes (Na^+^, Mg^2+^; K^+^, Ca^2+^, Cl^−^, NO^3−^, CO_3_^2−^; As^3+^, SO_4_^2−^; Mn^2+^; Pb^2+^; Zn^2+^, Cr^3+^, Co^2+^; Fe^3+^ and Cu^2+^)	[76]

**Figure 8:**
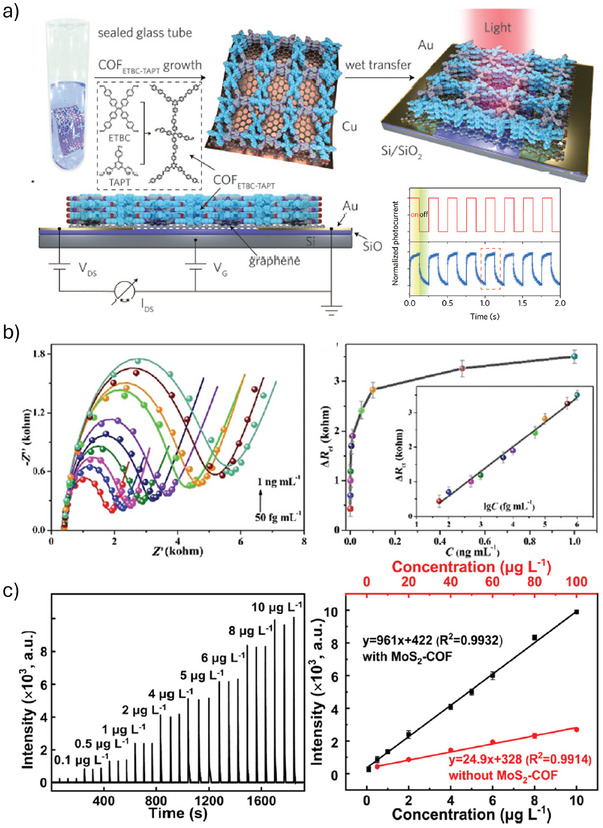
a) COF_ETBC–TAPT_ was oriented and grown on Cu‐supported CVD graphene in a sealed glass tube with a powder of COFs precipitated in the bottom of the reaction vessel. Photodetectors were fabricated by assembling a COF_ETBC–TAPT_‐graphene heterostructure with Au electrodes on a Si/SiO_2_ substrate. The area in dashed line is the chemical structures of COF_ETBC–TAPT_ and its monomers (top), side schematic view of a constructed COF_ETBC–TAPT_‐graphene photodetector and its measurement setup (bottom left), photoswitching performance under alternating dark and light illumination (V_G_ = 0, V_DS_ = 1 V, λ = 473 nm) (bottom right), Reproduced with permission.^[^ ^60^ ^]^ Copyright 2020, Wiley‐VCH; b) all EIS Nyquist plots for CAP detection at different concentrations (left), dependence of the ΔR_ct_ value on the CAP concentration. Inset: The calibration curves of ΔR_ct_ value versus logarithm value of CAP concentration (right), Reproduced with permission.^[^ ^73^ ^]^ Copyright 2021, Royal Society of Chemistry; c) temporal optical emission profile of nickel (left) and calibration curve obtained by the PVG‐DBD‐OES system based on a MoS_2_‐COF bifunctional supporter (right, Reproduced with permission.^[^ ^76^ ^]^ Copyright 2022, American Chemical Society.

Lei et al. developed advanced hybrid materials for high‐performance photodetectors by combining graphene's high charge carrier mobility with light‐harvesting COF comprising anthracene based monomers, yielding enhanced performance (photoresponsitivty: 0.54 ± 0.26 A W^−1^) compared to graphene and graphene functionalized with COF monomers (negligible photoresponse).^[^ ^89^ ^]^ Using submolecular‐resolution scanning tunneling microscopy (STM), the authors attributed the improved performance to the formation of a well‐ordered COF layer on graphene surface. To address the issue of limited carrier mobility of COFs, Lu et al. polymerized donor–acceptor monomers with photoelectric properties, such as tetra‐carboxaldehyde tetrabisphenylethylene (ETBC) and TAPT, into COF framework on graphene layer prepared through chemical vapor deposition (CVD) method.^[^ ^60^ ^]^ The photodetector fabricated with graphene‐COF_ETBC‐TAPT_ hybrid material showed excellent performance with a photoresponsivity of approximately 3.2 × 10^7^ A W^−1^ at 473 nm and a time response of ≈1.14 ms (**Figure** **8**a).

#### 4.1.2 Electrochemical Sensors

Electrochemical sensors are valued for their affordability, ease of manufacture, rapid analysis, and suitability for on‐site detection. They are particularly suitable for the detection of numerous types of analytes because they offer high sensitivity, selectivity, and the ability to detect a wide range of chemical and biological substances in various environments. However, they often lack the necessary sensitivity and selectivity. To enhance the sensitivity of electrochemical sensors, Qiao et al. developed a hybrid material consisting of NH_2_‐MWCNT‐COF and MoS_2_ nanosheets.^[^ ^39^ ^]^ NH_2_‐MWCNT‐COFs offer high conductivity, crystallinity, and surface area, while MoS_2_ nanosheets facilitate efficient electron transfer. Targeting sulfamerazine (SMR), a common antibiotic, the authors employed molecularly imprinted polymers (MIPs) to further improve detection capabilities. Sequential deposition of NH_2_‐MWCNT‐COF, MoS_2_, and MIP on a glassy carbon electrode (GCE) yielded a sensor with excellent SMR selectivity and reproducibility. The sensor exhibited a wide current response for SMR (concentration range between 3.0×10^−7^ to 2.0 × 10^−4^ mol L^−1^) and a low detection limit (1.1 × 10^−7^ mol L^−1^). Furthermore, this sensor was successfully applied for the determination of SMR in pork and chicken samples with recoveries of 86.0%–102.0%.

He et al. enhanced the sensitivity of electrochemical sensors by developing hybrid materials consisting of a COF, formed by TPAT and DHTA, GO‐NH_2_, and metallic Au NPs (Au@COF/GO‐NH_2_).^[^ ^73^ ^]^ To impart selectivity toward CAP, a common broad‐spectrum antibiotic, they utilized a specific aptamer ligand. GO‐NH_2_‐COF composites were first constructed by covalently linking COFs and GO‐NH_2_ by employing imine chemistry. The porous COFs facilitated the embedding of Au NPs through a straightforward impregnation‐reduction method. The resulting electrochemical impedance spectroscopy (EIS) sensor, with aptamers immobilized on gold electrodes, demonstrated ultrasensitive detection toward CAP with a limit of detection of 16.13 fg mL^−1^ (49.91 fM) and exceptional specificity and selectivity, even in the presence of high concentrations of interferents (Figure 8b).

Kong et al. developed a sandwich‐type electrochemical immunosensor for highly sensitive detection of the protein biomarker CYFRA21‐1, crucial in non‐small cell lung cancer diagnosis.^[^ ^90^ ^]^ They enhanced Ti_3_C_2_T_x_ stability and conductivity by modifying it with Au NPs (Au‐Ti_3_C_2_T_x_). Au‐Ti_3_C_2_T_x_ was used to capture primary antibodies (Ab_1_) against CYFRA21‐1 on a GCE electrode (GCE‐Au‐T Ti_3_C_2_T_x_ /Ab_1_), and to accelerate the electron transfer rate of the substrate. The sensor's second element comprised Au NPs‐doped COF polymer, where Au NPs improve the biocompatibility and conductivity of the pristine COF Tp‐Pa. This composite captured secondary antibodies (Ab_2_) and toluidine blue (TB) for signal amplification (Ab_2_‐TB‐Au‐COF), generating an enhanced electrochemical signal. The immunosensor exhibited remarkable sensitivity, stability, reproducibility, low cost, and environmental friendliness, with a wide current response for CYFRA21‐1 and a detection limit of 0.1 pg mL^−1^. This biosensing platform was successfully applied to analyze real serum samples, demonstrating the potential of Au‐ Ti_3_C_2_T_x_ and TB‐Au‐COF composites for biosensing.

#### 4.1.3 Optical Sensor

Microplasma‐based optical emission spectrometry (OES) is a technique used to analyze chemical compositions by exciting atoms or ions in a sample, causing them to emit light that can be detected and analyzed to identify the elements present.^[^ ^91^ ^]^ PVG has attracted significant interest because of its simplicity, ease of miniaturization, safety, and eco‐friendliness.^[^ ^92^ ^]^ However, improving PVG efficiency is crucial for achieving highly sensitive detection. To tackle this challenge, Wang et al. developed a MoS_2_‐COF Tp‐Pa composite that serves a dual purpose: it facilitates sample separation and enrichment due to the strong adsorption capacity of COFs for metal ions, and it enhances PVG efficiency through the formation of a heterojunction.^[^ ^76^ ^]^ This composite enables precise and sensitive detection of heavy metals, especially nickel ions, in environmental water samples using dielectric barrier discharge microplasma‐OES. The MoS_2_‐COF composite effectively enriches nickel ions while improving PVG efficiency, resulting in a detection range of 0.1−10 µg L^−1^ and a detection limit of 0.03 µg L^−1^ for nickel (Figure 8c). Compared to direct PVG methods, the composite exhibits significantly enhanced tolerance to coexisting interfering ions, leading to a 43‐fold reduction in detection limits.

### 4.2 Catalysis

#### 4.2.1 Hydrogen Evolution Reactions

Hydrogen is gaining recognition as a promising energy carrier, providing renewable, environmentally friendly, and high‐energy‐density advantages over current fossil fuel‐based technologies. Recently, COFs have emerged as a novel photocatalyst for HER due to their previously outlined advantages. Nevertheless, noble metal co‐catalysts like platinum are typically required in COF‐based photocatalysts to achieve a high hydrogen evolution rate, primarily because of the high recombination rate of photoinduced electrons and holes. Unfortunately, platinum's limited availability and high cost make it impractical for large‐scale applications. Hybridizing 2DMs with COFs leverages the excellent conductive properties, suitable band alignments, and catalytic potentials of 2DMs to improve charge separation, reduce recombination, and enhance the overall efficiency of hydrogen production.

##### Photocatalysis

**Figure 9:**
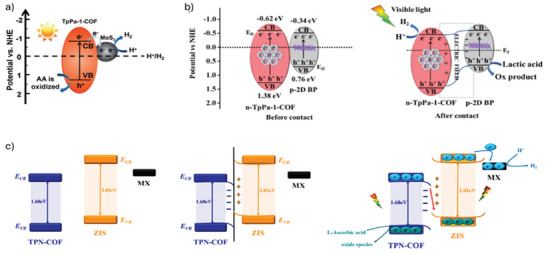
a) Schematic illustration of photocatalytic hydrogen evolution of MoS_2_/Tp‐Pa‐1‐COF composite photocatalysts under visible light irradiation, Reproduced with permission.^[^ ^80^ ^]^ Copyright 2019, Royal Society of Chemistry; b) schematic potential energy illustrations of the mechanism of photocatalytic hydrogen evolution for BP/Tp‐Pa‐1‐COF p‐n heterojunction, Reproduced with permission.^[^ ^87^ ^]^ Copyright 2023, Elsevier Science & Technology Journals; c) S‐scheme charge transfer process: (left) before contact, (middle) after contact, and (right) under light irradiation, Reproduced with permission.^[^ ^93^ ^]^ Copyright 2023, Wiley‐VCH.

In 2019, Zhang et al. designed and synthesized the first high‐performance noble‐metal‐free COF‐based photocatalyst for HER.^[^ ^80^ ^]^ They employed a conventional β‐ketoenamine‐based COF Tp‐Pa‐1 as a prototype and combined it with MoS_2_ to fabricate MoS_2_‐COF Tp‐Pa‐1 composites via the in situ growth of COF Tp‐Pa‐1 in an exfoliated MoS_2_ dispersion solution. Under visible light exposure and using ascorbic acid as the hole sacrificial agent, the MoS_2_‐COF Tp‐Pa‐1 composite, optimized with 3 wt% MoS_2_ loading, achieves a remarkable H_2_ evolution rate of 55.85 µmol h^−1^, with an apparent quantum efficiency of 0.76% at 420 nm (**Table** **4**). Compared to pure COF Tp‐Pa‐1 (1.72 µmol h^−1^), this composite exhibits a 32‐fold increase in H_2_ evolution rate and even surpasses Pt/COF Tp‐Pa‐1 (54.79 µmol h^−1^) with equivalent Pt loading (3 wt%). In the MoS_2_‐COF Tp‐Pa‐1 photocatalyst, Tp‐Pa‐1 exhibits notably visible light absorption capability. Consequently, upon visible light irradiation, electrons in COF Tp‐Pa‐1 are excited to the conduction band (CB) (**Figure** **9**a). These photogenerated electrons then undergo charge transfer and accumulate on the surface of MoS_2_. Subsequently, the separated electrons on MoS_2_ serve as reductants for H_2_ production, while the holes remaining in the valence band (VB) of COF Tp‐Pa‐1 are captured by ascorbic acid, being subsequently oxidized. Therefore, MoS_2_ not only acts as a co‐catalyst but also significantly enhances the transfer of photogenerated electrons from the COF to MoS_2_, thereby improving the separation of photogenerated charges and enhancing the H_2_ evolution activity of the composite.

**Table 4:** Performance of 2DM‐COF hybrid photocatalysts.

COF	2DM	Reaction	Rate [mmol h^−1^ g^−1^]	Amount of catalyst [mg]	Quantum efficiency	Refs.
Tp‐Pa‐1	MoS_2_	HER	5.5	10	0.76% at 420 nm	[80]
Tp‐Pa‐1	rGO	HER	11.98	10	7.53% at 450 nm	[67]
Tp‐Pa‐1	BP	HER	0.46	–	–	[87]
Tp‐Pa‐1	rGO and BP	HER	18.1		–	[88]
NTU‐BDA‐THTA	NH_2_−Ti_3_C_2_T_x_ MXene	HER	14.23	10	9.75% at 500 nm	[40]
Tp‐BD	g‐C_3_N_4_	HER	12.8	30	15.09 at 500 nm	[41]
TBTA	g‐C_3_N_4_	HER	11.7	5	–	[102]
ZIS‐TPN‐COF	Ti_3_C_2_T_x_ MXene	HER	4.01	–	–	[93]
TFPT and 4‐formylphenylboronic acid	g‐C_3_N_4_	HER	7.8	50	19.6% at 420 nm	[103]
CTF‐1‐G	WS_2_	HER	8.74	10	–	[44]
Tp‐Bpy‐Fe	Ti_3_C_2_Tx MXene	NRR	41.8 (µg h^−1^ mg_cat_^−1^)	–	43.1% at −0.5 V	[97]
Tp‐TAPT	g‐C_3_N_4_	NRR	0.22	50	–	[96]
Tp‐TAPT	g‐C_3_N_4_	CO_2_RR	0.6	20	–	[85]
Tp‐Pa‐1	Co‐1T‐MoS_2_	CO_2_RR	0.20	10	0.7% at 420 nm	[100]
Tp‐Ma	g‐C_3_N_4_	H_2_O_2_ production	880 µM h^−1^	25	–	[98]

In a similar approach, Zhang et al. employed COF Tp‐Pa‐1 to create a composite with rGO.^[^ ^67^ ^]^ The resulting rGO (5%)–COF Tp‐Pa‐1 exhibited a notable H_2_ evolution rate of 11.98 mmol g^−1^ h^−1^ under visible‐light irradiation. The authors demonstrated that the covalently functionalized rGO serves as an effective electron collector and transporter, enhancing the separation of photogenerated charges and facilitating the transfer of active electrons, thereby enhancing the overall H_2_ evolution activity.

An alternative strategy to solve the problem of the high recombination rate of photoelectron–hole pairs of COFs was developed by constructing a *p*‐*n* heterojunction between the COF Tp‐Pa‐1 and BP.^[^ ^87^ ^]^ The most effective sample (15% BP‐COF Tp‐Pa‐1) demonstrated a high hydrogen generation rate (456.7 µmol g^−1^ h^−1^) compared to all other heterostructures featuring non‐noble metal loading. Before the contact between 2D BP and COF Tp‐Pa‐1, the CB and VB edges of 2D BP are positioned between those of COF Tp‐Pa‐1 (Figure 9b). Upon loading p‐BP onto COF n‐Tp‐Pa‐1, a p‐n heterojunction forms at their interface due to the difference in Fermi level (E_f_) values. This leads to the migration of high‐energy electrons from COF n‐Tp‐Pa‐1 to p‐BP and holes from p‐2D BP to COF n‐Tp‐Pa‐1 until E_f_ aligns. An internal electric field is established from COF n‐Tp‐Pa‐1 to p‐BP. Consequently, the VB of BP increases with the E_f_, and the CB of COF Tp‐Pa‐1 decreases to E_f_. Synergistically, an internal electric field and diverse band potential in the p‐n heterojunction prompt electron migration from BP to COF Tp‐Pa‐1, reducing H^+^ for H_2_ production. Simultaneously, holes from the VB of COF Tp‐Pa‐1 transport to the VB of 2D BP, consumed by the selected hole sacrificial agent, ascorbic acid, ensuring photocatalytic H_2_ production progresses.

Zhao et al. have investigated the influence of linkage types on COF photocatalytic performance.^[^ ^40^ ^]^ They synthesized three COFs via acid‐catalyzed Schiff base reactions between Pa and TFB derivatives with varying hydroxy groups. These COFs exhibited diverse ratios of β‐ketoenamine to imine moieties, impacting their structure, and light absorption. Interestingly, surface area, structural order, and light absorption were not decisive factors in photocatalytic activity. Instead, a correlation was found between increased HOMO energy and enhanced photocatalytic performance with more β‐ketoenamine linkages. Furthermore, they successfully fabricated NH_2_−Ti_3_C_2_T_x_‐COF hybrids via covalent binding, showcasing efficient charge transfer and superior photocatalytic activity (hydrogen generation rate of 14.23 mmol g^−1^ h^−1^) and stability compared to non‐covalent heterostructures.

Li et al. developed a novel 1D 2D/1D composite heterojunction, g‐C_3_N_4_‐COF, by combining 1D β‐keto‐enamine‐based COF, formed by Tp and BD monomers, with 2D g‐C_3_N_4_.^[^ ^41^ ^]^ This unique heterojunction combines the advantageous properties of both 1D and 2D materials, resulting in superior photocatalytic performance. The enhanced carrier transfer and separation efficiency of g‐C_3_N_4_‐COF, attributed to its large interface contact, contribute to its remarkable photocatalytic activity. By incorporating 1D COF nanobelts, the composite extends its absorption range to 560 nm. The optimized g‐C_3_N_4_‐COF (10:1) achieves a H_2_ production rate of 12.8 mmol g^−1^·h^−1^ under visible‐light irradiation, surpassing COF and g‐C_3_N_4_ rates by 62 and 284 times, respectively. Additionally, g‐C_3_N_4_‐COF (10:1) exhibits an apparent quantum efficiency (AQE) of about 15.09% under 500 nm light, ranking among the highest reported for COF‐ or g‐C_3_N_4_‐based materials.

Li et al. introduced a novel ZnIn_2_S_4_ (ZIS)–COF Tp‐TAPA (TPN COF) S‐scheme heterojunction loaded with Ti_3_C_2_ MXene (MX) for efficient photocatalytic H_2_ evolution.^[^ ^93^ ^]^ The optimized sample, MX/TPN COF@ZIS (ZIS:TPN‐COF mass ratio of 3:2, with 2% MX), achieved an impressive H_2_ production rate of 4.01 mmol g^−1^ h^−1^ under visible light irradiation. The band structures suggest that ZIS acts as a reduction‐type photocatalyst, while TPN COF acts as an oxidation‐type photocatalyst (Figure 9c). Upon close contact, electrons from ZIS transfer to TPN‐COF at the interface, resulting in a positively charged ZIS surface and a negatively charged TPN COF surface, generating an interfacial electric field. Under visible‐light irradiation, electrons from TPN‐COF are excited to the conduction band and injected into the valence band of ZIS, while electrons from ZIS are excited to the conduction band and transferred to the cocatalyst MX. This process activates hydrogen evolution and oxidation of ascorbic acid by isolated photogenerated electrons and holes, respectively.

##### Electrocatalysis

Huang et al. demonstrated the potential of electrochemical water splitting using a hybrid of 2D COF‐42, formed by TFB and 2,5‐diethoxyterephthalamide (DETA), and Ti_3_C_2_T_x_ MXene.^[^ ^66^ ^]^ This combination not only creates porous channels at multiple scales for rapid electrolyte and electron transport but also exposes numerous catalytically active sites. The optimized Ti_3_C_2_T_x_‐COF nanoarchitecture exhibits outstanding HER properties, including a low onset potential of 19 mV, a small Tafel slope of 50 mV dec^−1^ (Pt/C 56.7 mV dec^−1^), and excellent long‐term durability (24 h) comparable to commercial Pt/C catalysts (**Table** **5**).

In their study, Yang et al. investigated a rGO‐COF hybrid loaded with ruthenium ions as an electrocatalyst for HER using nitrogen enriched monomers, piperazine (Pz), and cyanuric chloride (CC).^[^ ^94^ ^]^ In an alkaline aqueous medium, rGO‐COF‐Ru exhibits an onset potential close to 0 mV and an overpotential of 42 mV at 10 mA cm^−2^, surpassing Pt/C (52 mV) and demonstrating a low Tafel slope of 46 mV dec^−1^. The binding of Ru atoms to rGO‐COF and N atoms to the carbon skeleton redistributes electrons, increasing carrier density and reducing the catalyst's bandgap. Ru and Ru‐N sites within COF acted as central active sites for HER, while the incorporation of rGO improved conductivity.

#### 4.2.2 Oxygen Evolution Reactions (OER)

Solar‐driven PEC water splitting has emerged as a promising method for sustainable energy production.^[^ ^95^ ^]^ However, the OER on the photoanode often lags behind the HER on the photocathode due to sluggish kinetics. Similar to the challenges encountered in the HER, the application of pristine COFs has been constrained by inadequate surface charge transfer and excessive recombination of photogenerated charges at the interface between the photoanode and electrolyte.

**Figure 10:**
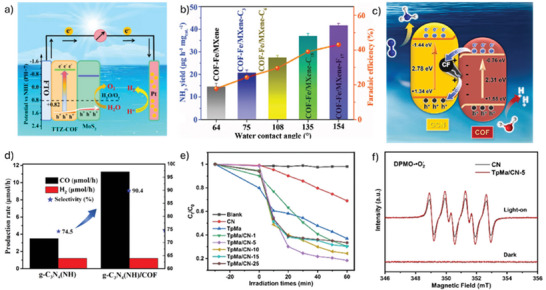
a) Schematic illustration of PEC water oxidation mechanism of 2D MoS_2_‐TTZ COF heterojunction under illumination, Reproduced with permission.^[^ ^69^ ^]^ Copyright 2022, Elsevier Science & Technology Journals; b) NRR performances of MXene‐COF‐Fe derivatives, Reproduced under the terms of the CC‐BY license.^[^ ^97^ ^]^ Copyright 203, Wiley‐VCH; c) the photocatalytic mechanism for NH_3_ evolution over GCNc‐COF, Reproduced with permission.^[^ ^96^ ^]^ with permission. Copyright 2024, Elsevier Science & Technology Journals d) photocatalytic yields and selectivity of CO and H_2_ for g‐C_3_N_4_ (NH) and g‐C_3_N_4_ (NH)/COF, Reproduced with permission.^[^ ^85^ ^]^ Copyright 2022, Elsevier Science & Technology Journals; e) time‐dependent photocatalytic degradation of TC by TpMa/CN‐5 photocatalyst, Reproduced with permission.^[^ ^98^ ^]^ Copyright 2022, Elsevier Science & Technology Journals; f) ESR signals of DMPO‐•O_2_^−^adduct over CN and TpMa/CN‐5 systems under visible light irradiation. Reproduced with permission.^[^ ^98^ ^]^ Copyright 2022, Elsevier Science & Technology Journals.

To address this challenge, Wang et al. devised a strategy to create a 2D heterojunction between a COF and MoS_2_.^[^ ^69^ ^]^ By using TAPT and TFPT monomers, they prepared a triazine‐based COF (TTZ COF) in the presence of a suspension containing delaminated MoS_2_. The photocurrent density achieved by the electrocatalytic NRR, a composite with MXenes was fabricated. MXene‐COF hybrid was functionalized with Fe (III) ions as an ideal co‐catalyst candidate among non‐noble metals for converting N_2_ into NH_3_. The MXene‐COF hybrid material functionalized with perfluoroalkyl chains (MXene‐F_17_‐COF‐Fe) with excellent hydrophobicity (water contact angle ≈154°) shows the maximum NH_3_ yield of 41.8 µg h^−1^ mg_cat_^−1^. and the highest Faradaic efficiency of 43.1% at −0.5 V versus RHE under ambient conditions (**Figure** **10**b). By repelling water molecules effectively, the enhanced hydrophobicity of MXene‐COF‐Fe inhibits HER path favoring NRR performance.

A different strategy was employed by Chen et al. where a composite between COF Tp‐TAPT, g‐C_3_N_4_ and nanocellulose derived carbon (CF) was fabricated through a two‐step thermal condensation.^[^ ^96^ ^]^ Initially, nanocellulose is utilized as the carbon precursor, which is combined with g‐C_3_N_4_ through hydrogen bonding to produce g‐C_3_N_4_‐nanocarbon fibers (GCNc). Subsequently, the net‐like COF is deposited onto the GCNc MoS_2_‐TTZ COF heterojunction reached 2.5 µA cm^−2^ at 1.23 V versus the RHE, representing an approximately twofold increase compared to pristine TTZ COF. Upon illumination, the MoS_2_’s energy band bending facilitates the separation of electron–hole pairs in TTZ COF (Figure 10a). Photogenerated holes accumulate in the VB of MoS_2_, driving water oxidation to produce oxygen. Meanwhile, electrons from TTZ COF migrate to the Pt counter electrode for HER.

#### 4.2.3 Nitrogen Reduction Reaction

In order to avoid the competition with HER and boost the efficiency of NRR, Du et al. decided to modulate the wettability of a COF, formed by Tp and 2,2′‐bipyridine‐5,5′‐diamine (Bpy) monomers, by introducing hydrophobic functional groups, such as alkyl and perfluoroalkyl chains.^[^ ^97^ ^]^ Besides, it boosts the surface to form the GCNc‐COF heterojunction via a Schiff base reaction. Based on the band structure analyses, the heterojunction formed between GCNc and COF can be classified as a type‐Z heterojunction. The potential mechanism for NH_3_ generation by GCNc‐COF is depicted in Figure 10c. When exposed to visible light, photoexcited electrons migrate from the VBs of GCN and COF to their respective CBs. The CF, serving as an electron mediator, additionally mitigates the recombination rate of photocarriers, enhances the interfacial migration of photoelectrons, and augments light absorption. The photoinduced electrons readily transfer from the CB of COF to that of GCN. Consequently, the N_2_ reduction reaction occurs on the GCN surface, leading to NH_3_ production, while protons are generated through water oxidation by the holes on the VB of COF. The photocatalytic nitrogen fixation rate of GCNc‐COF amounts to 238.1 µmol g^−1^ h^−1^, which is 8.92 times higher than that of pristine GCN (24 µmol g^−1^ h^−1^).

#### 4.2.4 Carbon Dioxide Reduction Reaction (CO_2_RR)

The depletion of fossil fuels and the escalating CO_2_ emission of greenhouse gases have precipitated urgent global energy and environmental crises. Consequently, extensive research efforts are underway to explore cost‐effective and durable photo/electro catalysts for CO_2_RR, aimed to address both environmental and energy challenges simultaneously. In 2015, Yaghi et al. introduced an optimized imine‐based COF configuration for CO_2_RR using as a cornerstone a cobalt porphyrin (Co‐TAP) and biphenyl‐4,4′‐dicarboxaldehyde (BPDA) monomers, forming an extended π‐conjugated system and reducing interlayer distance.^[^ ^99^ ^]^ This enhancement led to an increased carrier mobility and conductivity. At a potential of −0.55 V (vs RHE), the Faraday efficiency (FE) for CO_2_ reduction to CO reached 90%, with a conversion number of 290 000 and an initial turnover frequency (TOF) of 9400 h^−1^.

Ye et al. employed a novel approach by introducing nitrogen vacancies in g‐C_3_N_4_, known as defect engineering, to adjust the E_f_ gap between defective C_3_N_4_ nanosheets (C_3_N_4_ (NH)) and COF Tp‐TAPT.^[^ ^85^ ^]^ This strategy widened the E_f_ gap between g‐C_3_N_4_ (NH) and COF Tp‐TAPT, facilitating the recombination of invalid photogenerated carriers through an S‐scheme pathway. Among various heterojunction configurations, the S‐scheme heterojunction stands out for its arrangement of reduction and oxidation photocatalysts, with the reduction photocatalyst possessing a more negative CB and the oxidation photocatalyst having a more positive VB. This setup facilitates the preservation of charge carriers with optimal redox capabilities. The g‐C_3_N_4_ (NH)‐COF heterojunction, demonstrates a stable and highly selective CO (90.4%) generation rate of 11.25 µmol h^−1^ under visible light irradiation (Figure 10d). This rate is 45‐fold and 15‐fold higher than that of g‐C_3_N_4_ and g‐C_3_N_4_/COF, respectively.

Do et al. implemented a novel strategy involving single‐atom doping to enhance the activity, selectivity, and stability of photocatalysts, in particular 1T‐MoS_2_.^[^ ^100^ ^]^ They reported a hollow ketoenamine‐based COF Tp‐Pa‐1 decorated with Co‐doped 1T‐MoS_2_ as a functional photocatalyst (Co‐1T‐MoS_2_‐COF Tp‐Pa‐1) for the selective photoreduction of CO_2_ to CO. Through mitigating electron–hole recombination, enhancing visible light absorption, and facilitating CO_2_ coordination, activation, and sequestration, the Co‐1T‐MoS_2_‐COF Tp‐Pa‐1 hybrid demonstrated impressive photocatalytic CO_2_ reduction efficiency, reaching approximately 196 µmol g^−1^ h^−1^ of CO production. Comparatively, bare Tp‐Pa‐1 and Co‐1T‐MoS_2_ exhibited CO production rates approximately 1.23 and 1.6 times lower, respectively, than Co‐1T‐MoS_2_‐COF Tp‐Pa‐1. Furthermore, the Co‐1T‐MoS_2_‐COF Tp‐Pa‐1 hollow sphere hybrid showcased an outstanding 93% selectivity over HER.

#### 4.2.5 Photodegradation via Reactive Oxygen Species (ROS) Formation

The degradation of organic pollutants relies heavily on various ROS, including superoxide radicals (^•^O_2_^−^), hydroxyl radicals (^•^OH), hydrogen peroxide (H_2_O_2_), singlet oxygen (^1^O_2_), among others. Photocatalysis offers an effective solution by harnessing photogenerated electrons and holes to interact with molecular oxygen, facilitating ROS generation under illumination.

Xu et al. introduced a donor–acceptor conjugated COF known as Tp‐Ma, derived from the combination of Tp and Ma.^[^ ^98^ ^]^ This COF, when coupled with g‐C_3_N_4_, forms a type II heterojunction (g‐C_3_N_4_‐COF Tp‐Ma) aimed at enhancing photocatalytic efficiency. Specifically, the electron‐donor β‐ketoenamine and electron‐acceptor triazine units within the COF Tp‐Ma facilitate intramolecular charge transfer, thereby aiding in the separation of excitons. Additionally, the COF Tp‐Ma structure remains stable under light exposure due to the presence of irreversible β‐ketoenamine formation. The researchers optimized the g‐C_3_N_4_photocatalyst by varying the COF Tp‐Ma content, denoted as g‐C_3_N_4_‐X‐COF Tp‐Ma, where “X%” indicated the mass percentage of COF Tp‐Ma relative to g‐C_3_N_4_ (X = 1, 5, 10, 15, 25). The optimal g‐C_3_N_4_‐5‐COF Tp‐Ma photocatalyst demonstrates impressive performance in hydrogen peroxide production, reaching 880.494 µM h^−1^, which surpasses that of g‐C_3_N_4_ by 49 times. Additionally, serving as a multifunctional photocatalyst, the g‐C_3_N_4_‐5‐COF Tp‐Ma sample exhibits the highest efficiency in degrading tetracycline, with its degradation rate being enhanced by 2.3 and 4.3 times compared to COF Tp‐Ma and g‐C_3_N_4_, respectively (Figure 10e). By means of electron spin resonance (ESR) analysis, it was demonstrated that ^•^O_2_^−^ is the major reactive specie in the photocatalytic reaction of g‐C_3_N_4_‐COF Tp‐Ma, while hole (h^+^) and ^•^OH have a slightly positive contribution to the degradation process (Figure 10f).

Zhang et al. synthesized COF Tp‐Pa‐1 and combined it with MoS_2_ to create a 2D‐2D heterojunction composite.^[^ ^81^ ^]^ The photocatalytic performance of the resulting material was evaluated through the degradation of tetracycline (TC) and rhodamine B (RhB). Specifically, under simulated sunlight irradiation for 30 min, MoS_2_‐COF with a weight ratio of 20 (MoS_2_‐COF (20)) achieved a removal efficiency of about 98% for RhB, whereas pure MoS_2_ and COF only achieved 37.5% and 35.6%, respectively. Trapping experiments were conducted to investigate the photocatalytic mechanism by using different scavengers during RhB degradation by MoS_2_‐COF (20). The influence sequence of active radicals on RhB degradation was found to be h^+^ > ^•^OH > ^•^O_2_^−^. The generated ^•^OH and h^+^ radicals proved effective in oxidizing organic pollutants into smaller molecules such as CO_2_ and H_2_O.

Chen et al. combined the ROS production with the slow release of Ag^+^ of Ti_3_C_2_‐COF Tp‐Pa‐1‐Ag composite to obtain antibacterial effect against Staphylococcus aureus and Pseudomonas aeruginosa, reaching antibacterials rates of 99.60% and 99.78%, respectively.^[^ ^101^ ^]^

**Table 5:** Performance of 2DM‐COF hybrid electrocatalysts.

COF	2DM	Reaction	Tafel slope [mV dec^−1^]	Current density [mA cm^−2^]	Overpotential [mV]	Refs.
COF‐42 (TFB‐DETA)	Ti_3_C_2_T_x_ MXene	HER	50	–	19	[66]
Pz‐CC	rGO	HER	46	10	42	[94]
Pa‐TFB	Ni‐Ti_3_CNT_x_ MXene	HER	46	3.5	164	[104]
Co‐TAP‐Pa	Ti_3_C_2_ MXene	CO_2_RR	372	−9.33	–	[70]

### 4.3 Energy Storage

Both 2DMs and COFs are gaining significant attention for energy storage applications owing to their unique properties. Graphene offers a high surface area, excellent conductivity, and mechanical strength but suffers from restacking issues. MoS_2_ provides redox activity and layered structures for ion transport, yet it exhibits lower conductivity and mechanical stability. MXenes combine high conductivity, hydrophilicity, and surface modifiability but face environmental stability challenges due to oxidation. COFs exhibit high surface area, tunable porosity, and redox activity but face disadvantages such as low electrical conductivity and structural instability during cycling. Combining 2DMs with COFs for energy storage leverages the high conductivity and mechanical strength of 2DMs with tunable porosity and redox activity of COFs, potentially enhancing their overall performance and stability.

#### 4.3.1 Supercapacitors (SCs)

To address the issue of self‐agglomeration in GRMs, researchers have proposed cross‐linking them with COFs to create hybrid materials that demonstrate exceptional performance in supercapacitor applications. The efficacy of these SCs depends on factors like electron transfer rate and interlayer spacing within graphene, which can be modulated by employing specific COFs. For instance, in 2020, Chen et al. reported on the use of a rGO‐COF hybrid as electrode materials in SCs.^[^ ^72^ ^]^ A 2D imine‐linked mesoporous COF, synthetized by using Pa and 1,3,6,8‐tetrakis(4‐formylphenyl)pyrene (TFPPy) monomers, containing 2.3 nm mesopores and 2 nm thickness, acting as a spacer, was incorporated into rGO hydrogels through hydrothermal assembly. The COF growth on rGO surface effectively prevented nanosheet stacking, maximizing their surface accessibility to electrolyte ions. The optimized rGO‐COF hybrid, containing 20 wt% COF, demonstrated a gravimetric specific capacitance (321 F g^−1^), 60% higher than the capacitance observed for rGO (**Table** **6**).

**Table 6:** Electrochemical performance of different 2DM‐COF hybrids in supercapacitors.

COF	2DM	Device configuration	Electrolyte	Capacitance [F g^−1^]	Energy density [Wh kg^−1^]	Power density [W kg^−1^]	Capacitance retention upon cycling	Refs.
DAPy‐Tp	rGO	3 electrode system	1 M H_2_SO_4_	599 at 0.5 A g^−1^	20.1	250	88% after 20 000 cycles	[105]
TFPPy‐DA	rGO	3 electrode system	1 M H_2_SO_4_	321 at 1 A g^−1^	103	50	–	[72]
DAAQ‐Tp	rGO	Coin cells	PVA/H_2_SO_4_	452 at 0.2 A g^−1^	44	500	94 after 10 000 cycles	[111]
Ma‐TA	rGO	3 electrode system	6 M KOH	234 at 0.8 A g^−1^	14.6	400	86% after 3500 cycles	[107]
TAPT‐DHTA	a‐rGO	3 electrode system	1 M H_2_SO_4_	195 at 1 A g^−1^	12.2	500	84.6% after 5000 cycles	[112]
TAPT‐TFTA	a‐GO	3 electrode system	1 M H_2_SO_4_	295 at 1 A g^−1^	11.1	354	91.6% after 5000 cycles	[113]
TAPB‐TFB	GAs	Coin cells	1 M H_2_SO_4_	289 at 1 A g^−1^	25.3	400	92% after 50 000 cycles	[114]
DAAQ‐Tp	GA	Coin cells	1 M H_2_SO_4_	378 at 1 A g^−1^	30.5	700	88.9% after 20 000	[109]
DAAQ‐Tp	rGO	Coin cells	0.5 M H_2_SO_4_	269 at 0.5 A g^−1^	–	–	96% after 5000	[115]
DAAQ‐Tp	GO	3 electrode system	1 M H_2_SO_4_	429 at 1 A g^−1^	59.4	950	80% after 10 000	[79]
DAAQ‐Tp	MXene‐ 15	3 electrode system	1 M Na_2_SO_4_	290 at 0.5 A g^−1^	–	–	100% after 1000 cycles	[82]
DAAQ‐Tp	MXene	3 electrode system	1 M H_2_SO_4_	390 at 0.5 A g^−1^	27.5	350	88.9 after 20 000	[68]
Ma‐TFB	g‐C_3_N_4_	3 electrode system	6 M KOH	257.5 at 1 A g^−1^	45	659	86.05% after 2000	[110]

**Figure 11:**
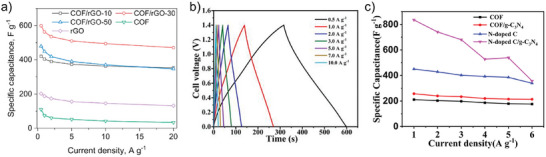
a) Gravimetric specific capacitance under different current densities for rGO, COF, COF/rGO‐10, COF/rGO‐30, COF/rGO‐50; Reproduced with permission.^[^ ^105^ ^]^ Copyright 2022, Elsevier Science & Technology Journals; b) GCD curves at various current densities; Reproduced with permission.^[^ ^68^ ^]^ Copyright 2020, Elsevier Science & Technology Journals; and c) specific capacitance for COF, COF/g‐C_3_N_4_, N‐doped C and N‐doped C/g‐C_3_N_4_ electrodes at different current densities, Reproduced with permission.^[^ ^110^ ^]^ Copyright 2022, American Chemical Society.

However, since the COF utilized lacks active redox units, it was speculated that employing redox‐active 2D‐COFs could further enhance their capacitive energy storage performance. Thus, Wei et al. hybridized rGO with a 2D‐COF synthetized with redox‐active monomers, Tp and 1,4‐diaminobenzene (DAPy). This method avoids rGO nanosheets agglomeration and a homogenously distributed COF over the rGO surface is obtained.^[^ ^105^ ^]^ The optimized hybrid, containing 30 wt% COFs (rGO‐COF‐30), exhibited a specific capacitance of 599 F g^−1^, significantly surpassing the specific capacitances of the pristine rGO (203 F g^−1^) and COF (110 F g^−1^) (**Figure** **11**a). Moreover, the incorporation of COF resulted in a capacitance retention of over 80% when the current density is increased by 40 times from 0.5 to 20 A g^−1^.

Enhancing the performance of carbon nanomaterials through heteroatom doping, such as nitrogen (N‐doped C), is known to boost conductivity, wettability, and capacitive performance compared to pristine carbon materials.^[^ ^106^ ^]^ COFs can be used as nitrogen atoms source to obtain N‐enriched carbon‐based nanocomposites for electrode materials. Sun et al. hybridized a triazine COF Ma‐TFB with GO, yielding a GO‐COF nanocomposite for SC applications.^[^ ^107^ ^]^ Both the COF and GO‐COF served as precursors for N‐doped carbon and N‐doped C‐rGO materials, respectively, through a simple carbonization step. Additionally, an ex situ approach involved the synthesis of COF separately, mixing it with GO, and then carbonizing them to produce N‐doped C‐rGO. These materials were evaluated as electrode materials for SCs, with N‐doped C‐rGO (in situ) demonstrating superior electrochemical performance, including high specific capacitance (234 F g^−1^) compared to pristine rGO (13.5 F g^−1^) and pristine COF (48.94 F g^−1^).

In recent years, the remarkable electrical conductivity and unique mechanical characteristics of graphene aerogels (GAs) have attracted tremendous attention in the field of energy storage.^[^ ^108^ ^]^ In this regard, Hu et al. employed an anthraquinone‐based COF Tp‐DAAQ featuring highly reactive positively charged sites to electrostatically self‐assemble with negatively charged GO, yielding a hybrid aerogel suitable for supercapacitor applications.^[^ ^109^ ^]^ Leveraging hierarchical porosity and a conductive network, the electrode demonstrated rapid charge transfer and efficient electrolyte ion transport. In a three‐electrode system, the synthesized redox‐active GA‐COF Tp‐DAAQ electrode exhibited a high specific capacitance of 378 F g^−1^ at 1 A g^−1^, nearly 24 times that of pristine COFs, along with a capacitance retention of 81.5% at 70 A g^−1^ and a cyclability with capacitance retention of 87.8% after 20 000 cycles.

In 2023, Xiuyan et al. utilized redox‐active COF DAAQ‐Tp and layered Ti_3_C_2_Tx MXene to fabricate MXene‐DAAQ‐COFs flexible composite film electrodes (CMFs).^[^ ^68^ ^]^ The synergistic effect between the ordered 1D pore structure of COFs and the intrinsic conductivity of MXene significantly enhanced electron transfer and ion migration rates, improving reaction kinetics for the CMFs. The galvanostatic charge–discharge(GCD) measurements demonstrated a specific capacitance of up to 390 F g^−1^ at 0.5 A g^−1^, nearly 12 times higher than that of pristine COFs, along with a ultrahigh capacitance retention of 81.3% at 10 A g^−1^ compared with 1 A g^−1^, indicating fast electrochemical kinetics (Figure 11b). The flexible CMF, requiring no conducting additives or binders, was directly employed in an all‐solid flexible supercapacitor, achieving a high energy density of 27.5 Wh kg^−1^ and a power density of 350 W kg^−1^, together with an excellent cyclability (capacitance retention of 88.9% after 20 000 cycles).

Abdelhamid et al. devised a novel one‐pot synthesis technique for fabricating g‐C_3_N_4_‐COFs hybrid, marking a pioneering endeavor in this domain. By conducting condensation reactions involving Ma and TFB, with and without g‐C_3_N_4_, they successfully synthesized COF and g‐C_3_N_4_‐COF materials.^[^ ^110^ ^]^ To enhance the electrical conductivity of the resulting 2DM‐COF hybrids, the researchers synthesized N‐doped carbon and N‐doped carbon/g‐C_3_N_4_ through the carbonization of COF and g‐C_3_N_4_‐COF, respectively. These carbonized materials exhibited a hierarchical porous structure comprising mesoporous‐macroporous regions. Notably, specific capacitance values amounted to 211 F·g^−1^ for COF, 257.5 F·g^−1^ for g‐C_3_N_4_‐COF, 450 F·g^−1^ for N‐doped carbon, and 835.2 F·g^−1^ for N‐doped carbon/g‐C_3_N_4_ (Figure 11c). Impressively, the asymmetric supercapacitor device incorporating N‐doped carbon‐g‐C_3_N_4_ demonstrated remarkable energy density (45.97 Wh·kg^−1^) along with high power density (659.3 W·kg^−1^).

#### 4.3.2 Batteries

##### Lithium‐Ion Batteries (LIBs)

**Figure 12:**
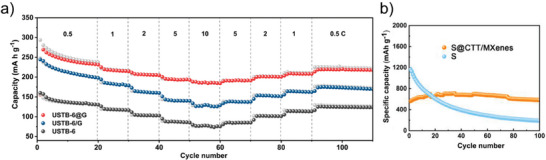
a) Cycling performances at 2 C and rate performances for USTB‐6, USTB‐6/G, and USTB‐6@, Reproduced with permission.^[^ ^116^ ^]^ Copyright 2022, Wiley‐VCH; b) cycling performance at 200 mA g^−1^ S@CTT/MXene, Reproduced with permission.^[^ ^119^ ^]^ Copyright 2022, Elsevier Science & Technology Journals.

Graphene nanosheets hybridized with a USTB‐6 COF were proposed by Jiang et al. as a new cathode material for LIBs. The latter was obtained through condensation between hexaazatrinaphthalene bearing six *p*‐formylphenyl groups (HATN‐CHO) and pyrene‐4,5,9,10‐tetraone (PTO‐NH_2_) moieties bearing high‐density redox‐active moieties.^[^ ^116^ ^]^ The addition of graphene to COF electrodes enhances electronic conductivity and facilitates COF nanosheet growth, aiming to improve battery performance. In particular, when the graphene‐COF hybrid is used as a cathode in LIBs, the device exhibits a specific capacity of 285 mA h g^−1^ at 0.2 C current density which is 3 times higher than pristine graphene (95 mAh g^−1^) (**Figure** **12**a). Moreover, even after 6000 charge–dischargecycles at 5 C, the graphene‐USTB‐6 nanosheet cathode maintained a capacity of 170 mAh g^−1^ (**Table** **7**).

**Table 7:** Electrochemical performance of different 2DM‐COF hybrids in batteries.

COF	2DM	Device configuration	Electrolyte	Capacity [mAhg^−1^]	Energy density [Wh kg^−1^]	Power density [W kg^−1^]	Cyclability	Cathode/Anode	Refs.
Lithium batteries									
TABQ‐PMDA	G	Coin cells	1 M LiTFSl	271 at 0.1 C	601	–	86% after 300 cycles	cathode	[127]
Ma‐TFB	g‐C_3_N_4_	Coin cells	1 M LiPF_6_	390 at 0.05 mA g^−1^	–	659	51% after 100 cycles	anode	[110]
PTO‐NH_2_ ‐HATN‐CHO	G	Coin cells	1 M LiTFSl	285 at 0.2 C	–	–	95% after 500 cycles	cathode	[116]
TFB‐Pa	MXene	Coin cells	1 M LiTFSl	780 at 1 C	–	–	300 cycles	anode	[62]
Lithium–sulfur batteries									
Tp‐guanidinium	MXene	Coin cells	1 M LiTFSl	1186 at 0.05 C	–	–	99.9% after 2000 cycles	–	[120]
TFB‐TAPA	MXene	Coin cells	1 M LiTFSl	935 at 0.05 A g^−1^	–	–	–	cathode	[119]
TFB‐Pa	MXene	Coin cells	1 M LiTFSl	1151 at 1 C	–	–	97.6 after 200 cycles	anode	[77]
DCB		Coin cells	1 M LiTFSl	1441 at 0.2 C	–	–	99.8% after 1000 cycles	cathode	[65]
TFFT		Coin cells	1 M LiTFSl	848	–	–	70% after 500 cycles	cathode	[117]
DPDN		Coin cells	1 M LiTFSl	1130 at 0.5 C	–	–	81.4% After 500 cycles	cathode	[118]
Sodium ion batteries									
TAPT‐ NTCDA		Coin cells	1.0 M NaClO_4_	109 at 0.1 A g^−1^	–	–	62% after 2000	cathode	[121]
Zinc ion batteries									
PDA‐TFB	MXene	Coin cells	0.2 M Zn(Ac)_2_	550 at 5 mA cm^−2^	–	–	–	cathode	[126]

tetramino‐benzoquinone (TABQ); pyromellitic dianhydride (PMDA); graphene (G); 1,4,5,8‐naphthalenetetracarboxylic dianhydride (NTCDA).

##### Lithium–Sulfur Batteries (Li–S)

Li‐S batteries offer several advantages over conventional LIBs including higher energy density, lower cost, and environmental friendliness. However, Li–S batteries face challenges such as limited cycle life due to the “shuttle effect” of lithium polysulfides (LiPSs), poor conductivity of sulfur and its final discharge products Li_2_S, and large volume change (>80%) during charge–discharge processes, leading to capacity fade and reduced performance over time.

Shi et al. devised a facile method for preparing a composite material as a sulfur host in Li–S batteries, where COF particles are enveloped by a GO covering film via a spray‐drying technique (GO/S‐COF).^[^ ^117^ ^]^ The polar groups on COF, given by the 2,4,6‐tris‐(4‐formylphenoxy)−1,3,5‐triazine (TFFT) and hydrazine monomers, efficiently adsorb lithium polysulfides through lithiophilic interaction, mitigating the “shuttle effect” caused by soluble LiPSs. Additionally, the GO outer layer wraps discrete sulfur particles, reducing the loss of active substances and enhancing the cathode's cycle stability. The GO/S‐COF cathode demonstrates a specific capacity of 1203.4 mAh g^−1^ at 0.2 C, 20% higher than pristine S‐COF. The cyclability test at 1 C for COF@GO/S and COF/S shows an initial specific capacity 848 and 709 mAh g^−1^, respectively, and a per‐lap attenuation rate after 500 cycles of 0.058 and 0.07%. By density functional theory (DFT) calculations, the authors demonstrated that the presence of COF in the hybrid material favors the adsorption properties and facilitates ion transport.

Meng et al. reported a novel approach that involves the in situ growth of porous phthalazinone‐based CTFs (P‐CTFs), through trimerization of 4,4′‐(1,5‐dione‐4,8‐diphenyl‐2,3,6,7‐tetraazaanthracene‐2,6‐diyl) dibenzonitrile (DPDN), onto rGO by the sulfur‐mediated cyclization of dinitrile monomers.^[^ ^118^ ^]^ This process yields rGO‐S/P‐CTF hybrids, which possess several advantageous features for enhancing Li−S battery performance. First, the nanoporous structure of P‐CTFs spatially traps sulfur species, while the covalent binding of sulfur and polar groups within P‐CTFs strongly attaches and adsorbs polysulfides, limiting their diffusion. Additionally, the presence of conductive rGO and semiconductive P‐CTFs facilitates faster electronic transportation and accelerates electrochemical processes. Consequently, the rGO‐S/P‐CTF cathodes exhibit significantly enhanced electrochemical performance, with a high initial specific capacity of 1130 mAh g^−1^ at 0.5 C and excellent capacity retention of 81.4% after 500 cycles, indicating minimal degradation per cycle.

Feng et al. designed a flexible and binder‐free cathode for Li–S batteries that consists of COF TFB‐TAPA‐derived N‐doped carbon (CTT) combined with Ti_3_C_2_ MXene.^[^ ^119^ ^]^ The electronically conductive porous N‐doped carbon‐MXene derived from COF acts as a sulfur host and efficiently limits the shuttle effect of LiPSs. To further enhance the safety and mitigate LiPSs diffusion, all‐solid‐state Li–S batteries are developed utilizing a polyethylene oxide (PEO)‐based solid electrolyte, known for its low LiPSs solubility and high safety profile. These all‐solid‐state Li–S batteries demonstrated outstanding safety characteristics and stable cycling stability, achieving a capacity of ≈584 mAh g^−1^ even after 100 cycles (Figure 12b).

Li et al. proposed a novel approach to enhance the performance of Li–S batteries by modifying the polypropylene (PP) separator with guanidinium‐based ionic‐covalent organic nanosheets (iCON) uniformly coated on Ti_3_C_2_ MXene nanosheets (Ti_3_C_2_‐iCON).^[^ ^120^ ^]^ The synergistic effects of Ti_3_C_2_ and iCON facilitate the efficient catalytic conversion of electrostatically trapped polysulfides, enhancing the electrochemical performance of carbon nanotube/sulfur (CNT‐S) cathodes. The modified separator demonstrated remarkable stability, with an average capacity decay of only 0.006% per cycle over 2000 cycles at 2 C. Even with a high sulfur content of 90 wt% and sulfur loading of 7.6 mg cm^−2^, the modified separator maintained high reversible capacity, areal capacity, and volumetric capacity.

##### Sodium‐Ion Batteries (SIBs)

SIBs represent a promising avenue in the realm of energy storage technologies, serving as a viable alternative to LIBs due to their cost‐effectiveness, as sodium is abundantly available and less expensive than lithium. Despite this merit, SIBs face challenges such as lower energy density compared to lithium‐ion counterparts, resulting in larger battery sizes for equivalent energy storage capabilities.

A triple structure regulation strategy is proposed by Lu et al. to enhance the performance of rGO‐polyimide COF hybrids as cathode materials for SIBs.^[^ ^121^ ^]^ This strategy involves morphology control, molecular design, and post‐synthetic vulcanization to optimize the COF structure. First, 2D COF nanosheets are formed with short channels through morphology control facilitated by π–π interactions between rGO and the COFs. Second, molecular design introduces more active sites in the COFs skeleton by using TAPT with a triazine ring instead of TAPA as the monomer. Lastly, post‐synthetic vulcanization transforms C═O bonds into more active C═S bonds, enhancing the activity of the COF surface (S‐TAPT). This triple regulation significantly improves the electrochemical properties of the rGO‐COF hybrid material, including specific capacity, rate ability, and ionic diffusion coefficient. The resulting rGO‐COF S‐TAPT hybrid exhibits outstanding performance as a cathode in SIBs, with a specific capacity of 109.3 mAh g^−1^ at 0.1 A g^−1^ and a retention of 68.6 mAh g^−1^ after 2000 cycles at a current density of 2.0 A g^−1^.

##### Zinc–Air Batteries (ZABs)

Zinc‐air batteries represent another innovative approach to energy storage, distinct from lithium‐ion technologies.^[^ ^122^, ^123^, ^124^, ^125^ ^]^ One of the most notable advantages of zinc–air batteries is their high energy density, owing to the abundance and low cost of zinc as well as the unlimited supply of oxygen from the air. The success of ZABs relies on the effective design of the air cathode, which involves complex bifunctional catalysts. Without proper material selection and structural design, the oxygen‐intermediate reaction can become sluggish, leading to reduced specific capacity and output power.

Zhu et al. reported the fabrication of a hybrid material comprising oxygen‐terminated Nb_2_CO_2_ MXene with COF‐LZU1 to be used as bifunctional oxygen catalytic performance in oxygen reduction reaction (ORR) and OER, as well as its potential application in ZABs.^[^ ^126^ ^]^ COF‐LZU1, with its fine pore structure, was embedded into Nb_2_CO_2_ MXene by a self‐assembly method to precisely restrict the flow of air and liquid (this is the so‐called mesoscopic confinement effect). Molecular dynamics simulations demonstrated that the presence of COF effectively controlled the concentration of O_2_ on the surface of Nb_2_CO_2_‐COF, thereby promoting an efficient and long‐lasting reaction. Analysis of the differential charge density and free energy revealed that Nb_2_CO_2_‐COF exhibited strong electron transport and catalytic activity, particularly on the COF side. When applied to ZABs, the Nb_2_CO_2_‐COF electrode demonstrated excellent stability (operating for more than 120 h) and power density (75 mW cm^−2^), suggesting its potential as a cathode material in ZABs.

### 4.4 Adsorption and Filtration

The rapid growth of industrial activities in recent decades has introduced a variety of pollutants into aquatic environments, posing significant challenges to environmental safety and public health.^[^ ^128^ ^]^ To address these issues, several water treatment methods have been employed including membrane purification, chemical precipitation, and adsorption.^[^ ^128^, ^129^, ^130^ ^]^ Among these, adsorption stands out as the most frequently employed, owing to its simplicity of operation, cost‐effectiveness, wide range of adsorbent materials, environmental friendliness, ease of scalability, and unique advantages when implemented on a large scale. In addition to adsorption, membrane separation technologies have played a crucial role in water purification for several decades, offering advantages such as high separation efficiency, low energy consumption, and ease of maintenance.

COFs, in view of their numerous and adjustable active sites, have found extensive use in both adsorption and filtration. Nevertheless, their chemical stability may be compromised under certain environmental conditions, and the staggered arrangement of COF layers can lead to pore blockage, reducing the accessibility of active sites and overall adsorption/filtration efficiency. Combining COFs with 2DMs offers compelling opportunities to enhance adsorption and filtration applications. 2DMs can offer structural reinforcement, improve mechanical stability, and potentially enhance mass transfer properties within COF‐based systems.

#### 4.4.1 Adsorption

##### Heavy Metal Ions and Radionuclides

Capacitive deionization (CDI) is an electrochemical technique that not only removes salt from water but also stores energy through either electrosorption or redox reactions. This method has become increasingly appealing as it offers a cost‐effective means of producing freshwater with minimal energy usage and high efficiency.^[^ ^131^ ^]^ Yamauchi et al. achieved this dual function by optimizing a heterointerface in a 2D MXene‐COF heterostructure through the assembly of a MXene (Ti_3_C_2_T_x_) with a β‐ketoenamine‐linked COF using DAAQ and Tp as monomers.^[^ ^132^ ^]^ The resulting heterostructure retains the unique 2D architecture and excellent electron conductivity of MXene while also possessing a large specific surface area and hierarchical porous structure similar to COFs. The outer COF layers prevent MXene nanosheets from restacking and oxidation, thereby safeguarding MXene from activity degradation during desalination/regeneration. Moreover, COF nanostructures with redox‐active DAAQ building monomers enhance Na^+^ ions capture. The MXene‐COF heterostructure demonstrates stable cycling performance over 100 CDI cycles, with a maximum NaCl adsorption capacity of 53.1 mg g^−1^ in oxygenated saline water (**Table** **8**). Notably, the MXene‐COF heterostructure outperforms most state‐of‐the‐art MXene materials, with a capacity nearly reaching 120 mAh g^−1^, highlighting the potential of MXene‐COF hybrids for electrochemical applications.

**Table 8:** Summary of 2DM‐COF hybrids for various pollutant adsorptions.

COF	2DM	Pollutants	Performance [mg g^−1^]	Refs.
Tp‐Pa‐SO_3_Na	GAs	Methylene Blue, Rhodamine B, Crystal violet	34;368; 328	[134]
Tp‐DAAQ	rGO	Organic solvents/oil	98.0–240.0	[115]
Ma‐Pa	PGO	Chlorogenic acid, caffeic acid	17.0; 17.2	[137]
Tp‐DAAQ	GO	U(VI)	220.1	[58]
TTF‐BN	rGO	U(VI)	521.6	[138]
Tp‐Pa	GO	U(VI), Eu(III)	1532.4; 886.4	[133]
Tp‐Pa	rGO	Pb^2+^	137.8	[139]
Ma‐TA	GO, g‐C_3_N_4_	Eosin, fluorescein	80–97.5	[86]
TAPB‐DVA	GO	naphthalene, 1‐naphthylamine, 1‐naphthol	211; 111	[135]
TAPB‐TFPT	MXene	fluoroquinolones	55‐148	[140]
TFTA‐TAPT	a‐GO	CO_2_	68	[113]

Graphene aerogel (GAs); 2,3,6,7‐tetra(4‐formylphenyl)tetrathiafulvalene (TTF), and 2,2′‐(biphenyl‐4,4′‐diyl)diacetonitrile (BN).

Li et al. synthesized a hybrid between COF Tp‐Pa and rGO, using them as CDI cathode for the selective removal of Pb^2+^ ions via an electrosorption process.^[^ ^74^ ^]^ In this scenario, rGO serves to enhance electrical conductivity, fostering the creation of an electric double‐layer capacitance (EDLC) that draws metal ions to the electrode surface. Conversely, the redox‐active COF Tp‐Pa can impart selectivity toward Pb^2+^ ions. The rGO‐COF Tp‐Pa‐hybrid properties resulted in >99.6% Pb^2+^ selective removal from complex solutions with the sorption capacities of 95 mg g^−1^ Pb^2+^ in single component solutions and 130 mg g^−1^ Pb^2+^ in multicomponent solutions.

Hu et al. developed a novel composite, 3D‐nanoflower GO‐COF Tp‐Pa‐1, that exhibited remarkable adsorption properties for the removal of U^6+^ and Eu^3+^ ions from aqueous solutions.^[^ ^133^ ^]^ A synergistic effect between GO and COF, resulted in a material with a high specific surface area (500.8 m^2^ g^−1^), abundant active functional groups, and excellent chemical stability. While COF provided abundant heteroatoms (O, N) as coordination sites for capturing U^6+^ and Eu^3+^ ions, GO provide chemical stability in acid conditions promoting the adsorption of radionuclides. The exceptional adsorption performance, 1532.35 mg g^−1^ for U^6+^ ions and 886.44 mg g^−1^ for Eu^3+^ ions at pH 6.5 and 298 K, was attributed to the unique skeleton structure and alleviated self‐stacking of GO layers facilitated by COF Tp‐Pa‐1, leading to significantly enhanced adsorption capacities.

##### Organic Pollutants

To address the issue of reduced adsorption capacity due to potential pore blockage in conventional COF materials, Kleitz et al. implemented a novel strategy involving the synthesis of ultra‐thin COFs using Tp and sodium 2,5‐diaminobenzenesulfonate (Pa‐SO_3_Na) as monomers.^[^ ^134^ ^]^ These ultrathin COFs offer increased exposure of active sites to targeted molecules within pollutant environments, thereby enhancing adsorption efficiency. Using a one‐pot hydrothermal approach, they integrated ultra‐thin COFs containing sulfonic acid moieties with GO serving as a surface template, resulting in the formation of a graphene‐COF aerogel (CGA) tailored specifically for dye adsorption. Remarkably, their findings demonstrated that while the pristine COF powder required 3 h to adsorb RhB dye, the CGA hybrid accomplished the same absorption in only 3 min. This substantial improvement in absorption capacity can be attributed to the unique 3D interconnected macroporous framework and well‐exposed adsorption sites facilitated by the CGA.

In 2020, Thomas et al. developed rGO‐COF aerogels tailored for highly effective adsorption of oil and organic solvents.^[^ ^115^ ^]^ The rGO‐COF hydrogel was formed employing organic monomers Tp and DAAQ in the presence of GO, which was reduced to rGO during the hydrothermal reaction. The aerogel exhibited low density, high conductivity, redox activity, and robust mechanical strength, showcasing exceptional absorption properties. Oil and sixteen organic solvents were targeted as pollutants, with absorption capacities surpassing 10 000 wt.% for oil and nearly all solvents. The 1:1 rGO‐COF aerogel displayed absorption capacities ranging from 98 to 240 times its own weight, outperforming many reported sorbents. This remarkable adsorption capability stemmed from the large specific surface area and superhydrophobic properties of COF Tp‐DAAQ. Moreover, the absorption capacity remained above 87% after 20 cycles, indicating the potential of rGO‐COF aerogels for efficient and recyclable oil clean‐up.

In their recent study, Qiu et al. synthesized a GO‐COF composite using 1,3,5‐tri(4‐aminophenyl)benzene (TAPB) and 2,5‐divinylterephthalaldehyde (DVA) as COF monomers, and GO as 2DM.^[^ ^135^ ^]^ The obtained GO‐COF TAPB‐DVA hybrid material was employed for the removal of small aromatic molecules, such as naphthalene, 1‐naphthylamine, and 1‐naphthol, from water. The adsorption capacity of GO‐COF for naphthalene (211 mg g^−1^), 1‐naphthalamine (110 mg g^−1^), and 1‐naphthol (98.2 mg g^−1^) improved significantly compared to individual components. Mechanistically, π–π interactions, hydrophobic interactions, pore‐size effects, were identified as primary adsorption mechanisms for naphthalene, while n–π electron‐donor–acceptor interactions, Lewis acid–base interactions, and H‐bonding were identified as primary adsorption mechanisms for 1‐naphthylamine and 1‐naphthol. Notably, GO‐COF TAPB‐DVA composite demonstrates excellent thermal and chemical stability across various pH environments. Furthermore, it exhibits high regenerative ability through ultrasonication in ethanol, enabling multiple reuses while maintaining satisfactory adsorption capacity.

##### Gas Adsorption

Although COF‐based materials have not been widely employed in gas adsorption, their potential in this realm is promising, especially for CO_2_ capture.^[^ ^136^ ^]^ At low temperatures, the CO_2_ adsorption process relies significantly on the pore architecture of COF‐based materials, being favored in pores smaller than 1 nm. Additionally, the functionalization of COFs with CO_2_‐coordination groups has proven effective in enhancing CO_2_ adsorption capacity also at low‐pressure conditions.^[^ ^136^ ^]^

Su et al. introduced a novel class of hybrid material for CO_2_ adsorption combining fluorine‐enriched COFs (COF‐F‐x) and aniline‐functionalized GO (a‐GO).^[^ ^113^ ^]^ The COF‐F‐x, chosen for its triazine and halogen groups, was synthesized using 2,3,5,6‐tetrafluoroterephthalaldehyde (TFTA) and TAPT as monomers. The authors synthesized various a‐GO‐COF‐F‐x materials, where x is the mass ratio of COF monomers input over a‐GO (x = m_a‐GO_/m_TAPT_ + m_TFTA_) in order to optimize the CO_2_ adsorption. These hybrid materials exhibit porous structures and intermolecular π–π interactions between GO and COF, facilitating rapid ion/electron transport and gas adsorption. The optimal CO_2_ adsorption capacities of a‐GO‐COF‐F‐2 at 273 and 298 K were found to be 67.8 and 52.8 mg g^−1^, respectively, surpassing those of pristine a‐GO (47.1 and 34.9 mg g^−1^). This enhancement in adsorption performance was primarily attributed to the adjustment of pore size and functional surface in the hybrid material.

#### 4.4.2 Filtration/Separation

Lu et al. harnessed the advantageous properties of GO, including its high hydrophilicity, chemical and mechanical stability, and straightforward surface modification to fabricate a GO/azine‐COF (GO‐ACOF) hybrid.^[^ ^141^ ^]^ Through a reaction conducted in an ampoule reactor, the authors synthesized a layer of ACOF on the surface of the activated GO membrane using TFB and hydrazine as monomers. The resulting GO‐ACOF membrane exhibited efficient removal of dyes (i.e., methyl blue and methylene blue) and salts (i.e., Na^+^, Ca^2+^, Mg^2+^, and Al^3+^ ions) from wastewater, along with antibacterial properties. This composite demonstrated high separation efficiency, exceeding 90%, attributed to molecular sieve separation, ion interaction, and the Donnan effect (**Table** **9**).

**Table 9:** The water permeation flux and rejection performance of 2DM‐COF hybrids‐based membranes.

COF	2DM	Feed	Permeation flux [L m^−2^ h^−1^ bar^−1^]	Rej.[%]	Refs.
TFB‐Pa	GO	Methylene blue, Congo red	59	9999.8	[145]
TFB‐hydrazine	GO	Methylene blue,	47	95‐99.2	[141]
TFB‐TA	prGO	Methylene blue Acid orange Rhodamine b	194	98	[78]
PBBA	GO	Methylene blue Chrome black T Congo red	310	99	[146]
DCB	GO	Methylene blue Alcian blue Congo red	226	99	[147]
Tp‐Pa	MXene	Congo red	563	99.6	[142]
TAPT‐Tp	Ti_3_C_2_T*_x_*	Trace organochlorine pesticides	–	99	[143]

Liu et al. developed dual‐layered MXene‐COF composite membranes by first constructing an intermediate COF Tp‐Pa layer on macroporous substrates, followed by the deposition of MXene nanosheets onto the COF layer.^[^ ^142^ ^]^ The COF Tp‐Pa layer, with its abundant pores and enhanced hydrophilicity, facilitated the formation of a well‐assembled MXene layer with a thickness as low as 8 nm. The ultrathin MXene‐COF hybrid membranes demonstrated remarkable separation properties, achieving a high permeance of 563 L m^−2^ h^−1^ bar^−1^ and an impressive rejection rate of 99.6% for congo red dyes. The enhanced water flux of the dual‐layered MXene‐COF composite membrane is attributed to the shortened pathways facilitated by the ultrathin MXene layer and the porous COF Tp‐Pa layer, while the heightened rejection is enabled by the narrow interlayer nanochannels formed between the well‐assembled MXene nanosheets.

Yamauchi et al. devised a monomer‐mediated strategy to hybridize 2D Ti_3_C_2_T_x_ MXenes with β‐ketoenamine‐linked COFs (TAPT‐Tp‐COF), forming well‐defined MXene‐COF heterostructures.^[^ ^143^ ^]^ By integrating 2D MXene nanosheets into the COF's framework, the resulting hybrid material exhibits synergistic properties from both components, addressing issues such as restacking of MXenes and aggregation of COFs while offering novel functionalities beyond what either component can achieve alone. MXene‐COF heterostructures were employed as novel coating adsorbents for solid‐phase microextraction (SPME) and demonstrated enhanced performance compared to pristine Ti_3_C_2_T_x_ MXene or COF TAPT‐Tp in capturing organochlorine pesticides (OCPs).

The performance of laminate membranes composed of 2DMs is intricately linked to their nanoscale structures, including interlayer spacings and supporting substrate pore sizes. Inspired by the concept of rebar graphene,^[^ ^144^ ^]^ Chen et al. proposed to reinforce prGO networks with a rigid nanospacer, such as a 2D COF‐LZU1, creating a robust prGO‐COF laminate membrane on nylon substrate.^[^ ^78^ ^]^ Intercalating COF into prGO laminates increases interlayer spacing and provides direct transfer channels, reducing water transfer resistance, while also enhancing the self‐supporting capacity of prGO networks on substrates with large pores. This strategy results in a 27‐fold increase in water permeance (≈200 L m^−2^ h^− 1^ bar) compared to pristine prGO membranes without sacrificing rejection rates (above 98%) to organic dyes (i.e., methylene blue, orange II sodium salt, and RhB). Furthermore, prGO‐COF membranes exhibit remarkable deformation resistance under compressive stress up to 5 bar with significantly enhanced water permeance.

### 4.5 Other Applications

#### 4.5.1 Anticorrosion

According to the National Association of Corrosion Engineers (NACE) International Institute, the global cost of corrosion is estimated at US$2.5 trillion. Moreover, corrosion poses risks to human and animal health, as heavy metals can accumulate in soil and water, which is increasingly recognized as a global concern.^[^ ^148^ ^]^ The application of COFs in anticorrosion has garnered interest because of their stability in harsh environments and their ability to act as nanocontainers for inhibitors. This facilitates the creation of smart coatings with self‐healing properties, which effectively suppress surface corrosion. However, their porous structure can allow corrosive agents to penetrate, potentially diminishing their shielding effectiveness. Combining COFs with 2DMs can enhance anticorrosion applications by providing additional structural reinforcement, improving barrier properties, and further enhancing the stability and effectiveness of the coatings.

Drawing inspiration from natural nacre, Arjmand et al. devised an anticorrosion material for metallic surfaces, through the in situ growth of COF Ma‐TA on MoS_2_.^[^ ^57^ ^]^ This MoS_2_‐COF hybrid serves multiple purposes: 1) the robust porous COF acts as a nanocontainer for europium, an inorganic inhibitor that can be released upon pH changes, 2) the dispersibility of MoS_2_ is enhanced through covalent grafting of COF, and 3) the barrier protection efficiency is improved by utilizing MoS_2_. The resulting nanocomposite coatings exhibited impressive total resistance of nearly 50 kΩ cm^2^, maintaining high impedance values (10^11^ Ω cm^2^) even after 77 days of immersion, surpassing that of pure epoxy. Additionally, salt spray analysis revealed minimal rust and corrosion product formation after 31 days in the filled nanocomposite coating (**Table** **10**).

**Table 10:** Electrochemical performance for 2DM‐COF hybrids in anticorrosion applications.

COF	2DM	Total resistance [kΩ cm^2^]	Impedance [Ω cm^2^]	Absence of corrosion product days	Cathodic delamination radius [nm]	Adhesion loss [%]	Refs.
TA‐Ma	MoS_2_	50	10^11^ (77 days)	31	3.41	24%	[57]
Tp‐Pa	GO	–	8.6 × 10^8^ (60 days)	45	–	–	[150]
TA‐Ma	GO	–	6.8 × 10^9^ (60 days)	15	–	34%	[149]
TA‐Ma	MXene	–	10.6 (77 days)	28	–	22%	[75]

In 2023, Chen et al. designed a robust anti‐corrosive epoxy coating incorporating a nano‐hybrid filler. The fabrication of the nano‐hybrid filler comprises two steps, first the surface of GO underwent amination with APTES resulting in amino‐functionalized GO (FGO), followed by in situ growth of porous COF Ma‐TA.^[^ ^149^ ^]^ While GO reduced the propensity for delamination in the epoxy coating by blocking micro‐cracks, defects, and the diffusion of corrosion ions at the interface, COF, with a micropore diameter, impedes the movement of oxygen, water molecules, and hydroxyl ions at the delamination interface, resulting in decreased activity in cathodic ORR and HER. Remarkably, the epoxy coating with 1 wt.% FGO‐COF exhibited the highest long‐term impedance resistance (6.8 × 10^9^ Ω cm^2^ after 60 days of immersion in seawater) and the lowest delamination area ratio (≈34% after 120 h).

Taking advantage of the barrier properties of Ti_3_C_2_ MXene and the porous structure of COF Ma‐Ta, Arjmand et al. developed a customized hybrid framework combining an imine‐based COF and MXene nanosheets as an anticorrosion coating for metal surfaces.^[^ ^75^ ^]^ This approach prevented oxidation and restacking of Ti_3_C_2_ MXene while enhancing its surface area (i.e., from 27 m^2^ g^−1^ of pristine MXene, to 128 m^2^ g^−1^ of MXene‐COF hybrid). The prepared hybrid material is suitable for hosting a high amount of inhibitors such as zinc and glutamate molecules. The controlled release of inhibitors (983 ppm zinc at acidic pH) for up to 96 hours resulted in 88% corrosion inhibition of the metal surface. Furthermore, incorporating this novel nanoplatform into an epoxy coating conferred self‐healing properties, leading to over 200% improvement in active corrosion prevention compared to unfilled coatings. The epoxy nanocomposite exhibited a low resistance of 10.64 Ω.cm^2^ after 11 weeks in a 3.5 wt% saline solution, as well as a low adhesion loss of only 22.5% on the metal surface.

#### 4.5.2 Flame Retardant Materials

Due to growing concerns about the environmental impact and bio‐accumulation of halogenated flame retardants, there is increasing interest in phosphorus and nitrogen‐containing alternatives for flame retardation in polymeric materials. Phosphorus‐nitrogen (PN) synergistic flame retardant systems have been extensively studied for their high efficiency.^[^ ^151^ ^]^ On the one hand, BP nanosheets, a type of phosphorus‐based flame retardant, have shown effectiveness in both gas and condensed‐phase flame retardation mechanisms, accelerating polymer carbonization and char formation.^[^ ^152^ ^]^ On the other hand, CTFs have also emerged as promising flame retardants due to their unique structure, abundant nitrogen element, and high stability in organic media.^[^ ^153^ ^]^

Inspired by the properties of BP and CTF nanosheets, Hu et al. integrated them into a 2DM‐COF Ma‐CC hybrid to explore synergistic effects on flame retardancy in epoxy resin.^[^ ^42^ ^]^ Functionalized BP nanosheets with NH_2_ groups were synthesized and combined with CTF through a solvothermal process to create BP‐NH_2_‐CTF nanohybrids. These nanohybrids significantly reduced smoke production and heat release in epoxy‐BP‐NH‐CTF nanocomposites, in particular, the addition of 2 wt% BP‐NH‐CTF results in a maximum decrease in peak heat release rate (61.2%) and total heat release (44.3%), accompanied by a substantial improvement limiting oxygen index performance (29.0%) and the reduction of the amount of toxic carbon monoxide and flammable volatile products. Furthermore, the 1 wt% EP‐BP‐NH‐CTF nanocomposites showed considerable improvements in their mechanical properties, including a notable 67.1% enhancement in storage modulus, accompanied by an increase in the glass transition temperature.

#### 4.5.3 Composites with Thermal, UV‐Shielding, and Mechanical Properties

Najmi et al. introduced a novel approach by synthesizing a nacre‐inspired structure composed of MoS_2_ nanosheets decorated with COF nanoparticles.^[^ ^59^ ^]^ The COF was produced through a condensation reaction using cost‐effective Ma and TA monomers. This combination resulted in nitrogen‐rich and conjugated COF structures, complementing the inorganic nature of MoS_2_. The resulting epoxy nanocomposites exhibited remarkable UV‐shielding, mechanical, and thermal properties. Remarkably, even with a small loading of the nanohybrid (0.15 wt%), significant enhancements were observed in the storage modulus (33%), cross‐linking density (230%), glass transition temperature (42%), and tensile strength (225%) of the coated materials. Thermal analysis revealed a 12% lower temperature in the nanocomposite coatings compared to pristine epoxy coatings after exposure to flame for 3 min, indicating their efficacy as thermal barriers. Moreover, weathering tests demonstrated minimal color change in the nanocomposites after prolonged UV light exposure, indicating their durability.

Ramezanzadeh et al. devised a composite material utilizing MXene and COF Ma‐TA. Initially, they treated MXene nanosheets with silane functional groups, resulting in Si‐MX, followed by the synthesis of a novel COF directly on the surface and edges of the nanosheets, forming Si‐MX‐COF.^[^ ^71^ ^]^ The composite served as an additive in multifunctional epoxy coatings, where COF enhanced the compatibility of MXene nanosheets with the coating, leading to improved dispersion and heightened levels of hardness and stiffness. Specifically, the glass transition temperature, storage modulus, and tensile strength were boosted by 28 °C, 30%, and 80%, respectively. Moreover, the loaded nanocomposites exhibited minimal color change after UV exposure, indicating their durability.

## 5 Conclusion and Future Outlook

The hybridization of 2DMs with COFs represents a cutting‐edge approach yielding advanced materials with synergistic properties, as summarized in Tables 1, 2. This strategy leverages the unique characteristics of both 2DMs, and COFs, potentially overcoming individual limitations and enhancing overall performance in various applications. However, while the concept of 2DM‐COF hybridization is promising, several challenges must be addressed to unleash the full potential of these hybrids.

### 5.1 Pristine COFs

One significant issue is the choice of COFs’ monomers and the corresponding type of linkages. Imine COFs notoriously suffer from limited stability when exposed to harsh conditions like strong acids, bases, or redox agents, and are less thermally stable compared to boronate‐ester‐linked counterparts, limiting their industrial applications in catalysis and gas storage. Meanwhile, 2D p‐conjugated COFs are promising for electronic and energy devices due to their tunable semiconducting properties. However, even with (photo)electroactive units like porphyrins and pyrenes, 2D COFs show limited in‐plane π‐conjugation due to the polarization of the C═N bond, restricting their effectiveness. In this case, different synthetic routes should be explored for example comprising the imine‐to‐quinoline transformation strategies. Another limitation is determined by the limited crystallinity of COFs, and in such a case the development of novel methodologies, e.g., based on advanced processing techniques employed during the growth of the COFs, can offer major enhancements in crystallinity with the ultimate goal of developing single‐crystal COFs.

### 5.2 Pristine 2DMs

There are >2000 possible 2D materials with a wide range of, often superlative, properties. Experimental studies have only just scratched the surface, with no more than 100 2DMs studied to date. By employing a materials genomics approach, scientists can leverage theoretical modeling and simulations to perform a comprehensive screening of a wide range of 3D layered materials, identifying potential precursors for innovative 2DMs with versatile properties.

### 5.3 Fabrication of 2DM‐COF Hybrids

Hybridization strategies such as solvothermal in situ synthesis of COF on the surface of 2DM; hydrothermal in situ synthesis of 2DM on the surface of COF, and ex situ synthesis of both components followed by their hybridization via blending have been explored, but each comes with its own set of complexities and limitations. Future research should focus on developing more robust and versatile synthesis methods that ensure uniform integration and stable performance of 2DM‐COF hybrids. Exploring new combinations of 2DMs and COFs with tailored properties could also lead to hybrids with enhanced functionalities. Future advancements in 2DM‐COF hybrids should emphasize enhancing the understanding of interfacial interactions and their impact on hybrid performance. This entails conducting comprehensive mechanistic studies to unravel charge transfer dynamics, ion transport mechanism, and chemical stability at the 2DM‐COF interface, leveraging state‐of‐the‐art in situ spectroscopy and advanced electron microscopy techniques. In parallel, efforts should prioritize device integration, emphasizing scalable synthesis methods tailored for industrial applications. These hybrids should be incorporated into innovative architectures, such as flexible and wearable devices designed for energy storage, sensing, and environmental monitoring. Exploring eco‐friendly synthesis methods, with reduced dependence on harmful solvents and energy‐intensive processes, will be equally crucial in view of sustainable development. Theoretical and computational studies will play a pivotal role in guiding the design of hybrids with tailored properties, enabling the prediction of optimized bandgaps, adsorption sites, and catalytic performance for specific applications. Lastly, addressing the durability and stability of 2DM‐COF hybrids remains critical for their long‐term usability. Ensuring robustness under harsh conditions – such as high temperatures, extreme pH environments, or extended operational cycles – will be essential for their viability in real‐world applications.

### 5.4 2DM‐COF Hybrids for Sensing Applications

The field of sensors should aim at overcoming the limited carrier mobility and signal interferences from coexisting analytes. This will be achieved, in the first case, by exploring new combinations of COFs with 2DMs exhibiting high electrical conductivity or by designing novel highly conductive COFs. In the second case, selectivity can be achieved by decorating the hybrid materials with ad hoc (supra)molecular receptors tailored for the analyte of interest. Additionally, the integration of machine learning and artificial intelligence for data analysis and optimization is expected to revolutionize the field.

### 5.5 2DM‐COF Hybrids for Catalysis Applications

In order to boost the catalytic performance of 2DM‐COF hybrids, researchers are likely to focus on addressing current challenges such as suppressing the charge carrier recombination as well as improving the stability and scalability of hybrids‐based catalysts. Efforts will aim at reducing dependence on costly noble metals such as platinum and enhancing overall efficiency through novel material combinations and structural modifications (e.g., defect engineering), to boost charge separation and catalytic performance. Future efforts may also focus on advanced synthetic techniques to tune the energy levels of both COFs and 2DMs to completely suppress charge carrier recombination and optimize catalytic performance. Moreover, exploring the feasibility of 2DM‐COF hybrids in real‐world applications, including large‐scale water splitting and pollutant degradation, will be crucial for transitioning these materials from laboratory innovations to practical implementations in sustainable energy and environmental remediation technologies.

### 5.6 2DM‐COF Hybrids for Energy Storage Applications

In the field of energy storage, future research on advancing the synthesis methods of both COFs and 2DMs to achieve precise control over the hybrid structure at the nanoscale could optimize material properties such as ionic and electrical conductivity, porosity, surface area, and chemical stability. Incorporating redox‐active moieties into COFs to improve their electrochemical activity, as demonstrated in recent studies, could further boost energy storage capacities in both SCs and batteries. Moreover, improving the chemical stability of COFs to ensure their long‐term durability under operational conditions will be essential for transitioning these materials from laboratory settings to commercial applications.

### 5.7 2DM‐COF Hybrids for Adsorption and Filtration Applications

A major challenge that can reduce the efficiency of 2DM‐COF hybrids in adsorption and filtration applications is the potential for pore blockage in COFs due to the staggered arrangement of layers, which can impede the accessibility of active sites. Although 2DMs can provide structural reinforcement and potentially improve mass transfer, the integration process must be carefully controlled to avoid exacerbating pore blockage. Future research directions will also focus on tailoring the 2DM and COF architectures and functionalities to achieve selective adsorption of specific pollutants, such as heavy metals, organic contaminants, and gases. This could involve precise control over pore size distribution, surface chemistry modifications, and the integration of functional groups that enhance target molecule interactions.

### 5.8 Advancing Comparative Evaluations

A critical gap in current research on 2DM‐COF hybrids is the frequent lack of systematic comparisons between the physicochemical properties and performance of pristine materials and their hybrid counterparts. While hybrid materials often exhibit enhanced performance, the underlying mechanisms driving these improvements are not always thoroughly investigated or reported. Researchers often focus on measuring the performance of hybrids without adequately documenting the baseline properties and performance of the pristine materials. Addressing this issue is essential for correlating the properties of individual components with the enhanced functionalities observed in hybrids. Future research should emphasize comprehensive evaluations of both pristine and hybrid materials, aiming to elucidate why specific synergies arise. Such studies would advance the rational design of hybrids by providing deeper insights into the interplay between the structure, properties, and performance of 2DMs, COFs, and their combinations. This approach will be critical in guiding the development of next‐generation hybrid materials optimized for specific applications

Overall, in view of the dynamic nature of this research field, future advancements in 2DM‐COF hybrids‐based materials will continue to drive innovations in sensing, catalysis, energy storage, environmental remediation, and filtration technologies, shaping sustainable solutions for diverse disruptive applications.

## Conflict of Interest

The authors declare no conflict of interest.
